# Identifying the determinants of hospital readmission in dementia patients – A retrospective cohort study using electronic healthcare records in England

**DOI:** 10.1371/journal.pone.0351331

**Published:** 2026-07-15

**Authors:** Bria Browne, Imogen Rogers, Khalid Ali, Naji Tabet, Elizabeth Ford

**Affiliations:** 1 Centre for Dementia Studies, Brighton and Sussex Medical School, Brighton, United Kingdom; 2 Department of Primary Care and Public Health, Brighton and Sussex Medical School, Brighton, United Kingdom; 3 Department of Clinical and Experimental Medicine, Brighton and Sussex Medical School, Brighton United Kingdom; University of Oxford, UNITED KINGDOM OF GREAT BRITAIN AND NORTHERN IRELAND

## Abstract

**Background:**

People living with dementia are at an increased risk of multiple hospital admissions due to acute illness, underlying vulnerability, progression of behavioural symptoms of dementia, or breakdown of care provision. Reducing readmission rates is a key clinical priority. This study used linked electronic health records from general practice and hospitals in England, to investigate the determinants associated with hospital readmissions and estimate the mortality risk of older adults with dementia within one year of hospital readmission.

**Methods:**

A retrospective cohort study using Clinical Practice Research Datalink and linked data. Patients were included if they were > 65y, diagnosed with dementia, and had a recorded acute general hospital admission. Outcome variables were readmission to hospital within 180 days of discharge, and in the readmitted group, mortality within one year of readmission. Demographics, long term conditions, polypharmacy, antipsychotic medication, medication review, post-discharge primary care appointments, and living in residential care were examined as determinants using stepwise logistic regression for readmission, and Cox regression for survival models.

**Results:**

30,527 patients were included, median age 84y (62.7% female), with 12,351 (40.5%) readmitted within 180 days of discharge. Being male, having multiple long-term conditions, having a medication review or attending a primary care appointment post-discharge were positively associated with readmissions, while Alzheimer’s type dementia, having ‘Other’ ethnicity, and living in residential care were negatively associated. We found a significant effect on mortality risk of age, sex, type of dementia, having two or more long-term conditions, body mass index, antipsychotic medication, polypharmacy, and living in residential care.

**Conclusion:**

The findings of this cohort study suggest that biological, psychological, and social factors interact to influence hospital readmissions in older adults with dementia. Multiple long-term conditions emerged as the strongest determining factor for readmission, while age was important in survival. Future research should assess psychosocial factors such as social support, caregiver burden, and mental well-being. These findings have important implications for strengthening community-based healthcare for adults with dementia, to reduce hospital readmission.

## Introduction

More people with dementia are living longer with multiple chronic conditions [1–3], requiring complex care and increasing the risk of multiple hospital admissions. Previous research has demonstrated that 30-day hospital readmissions in older adults with dementia are related to accumulating long-term conditions including cardiovascular disease [4] pneumonia [5], recurrent urinary tract infections, unmanaged behavioural and psychological symptoms of dementia (BPSD) [6], adverse drug reactions [7] and poor healthcare staff coordination [8]. The reasons for a subsequent hospital admission often differ from those of previous admissions, suggesting a heightened severity of clinical need and increased vulnerability during care transitions [9,10]. Hospital readmissions within 30 days are commonly used as performance indicators within healthcare systems, including the NHS [11,12]. However, this threshold is mainly policy-driven and may not adequately reflect clinical recovery trajectories, particularly among older adults with dementia.

Older adults living with dementia frequently experience multimorbidity, cognitive impairment, and reduced physiological reserve, which may prolong recovery following acute illness [13,14]. This extended period of generalised vulnerability is described as post-hospitalisation syndrome, in which individuals remain at a heightened risk of adverse health events following hospital discharge, therefore increasing the likelihood of subsequent readmission [15]. For people living with dementia, this vulnerability may extend beyond the conventional 30-day window. Evidence demonstrates substantially higher readmission rates at 180 days compared with 30 days in dementia populations, with elevated rates of readmission and mortality persisting for up to one year following discharge [16,17]. As such, shorter hospital readmission windows may underestimate the sustained post-hospital discharge instability experienced by people living with dementia. Furthermore, the choice of readmission timeframe has important implications for how this risk is understood, measured, and interpreted among this population. While reducing readmission rates is a key clinical priority, understanding its association with mortality may help identify individuals who may benefit from earlier palliative care involvement and personalised healthcare planning, ensuring that care aligns with individual needs and preferences.

Much of the existing research on the clinical determinants of hospital readmissions in dementia is based in the United States of America (USA), which operates under a different healthcare system than the United Kingdom (UK) [6]. Identifying determinants of hospital readmissions and subsequent mortality risk is essential for understanding the healthcare risks and needs of older adults with dementia in the UK. The UK has an established model of primary health care (known as general practice (GP)) via the National Health Service (NHS) (coverage ≈ 98% of the UK population), and data extracted from linked general practice and hospital patient records, which collectively can give a longitudinal view of patients’ health and disease [18–20]. This study used linked electronic health records to investigate demographic and clinical determinants associated with acute general hospital readmissions within 180 days of an index hospitalisation among older adults living with dementia, and to identify the determinants associated with mortality within one year of readmission.

## Methods

### Study design

A retrospective cohort study was developed from the Clinical Practice Research Datalink GOLD database.

### Data source – The clinical practice research datalink

Data from this study was extracted from anonymised primary care electronic health records via the Clinical Practice Research Datalink GOLD database (CPRD) [21]. CPRD is a population-based database of electronic health records from a UK-wide network of over 2,400 primary care practices, which include 65 million patients [22]. Within these 65 million primary care patients, 19 million are currently registered as active patients with at least 20 years of data [22]. Characteristics of the electronic health records include medical diagnoses, referrals to specialists and secondary care, chronic conditions, interventional procedures performed in primary care, and medication prescribed in primary care, which up until 2020, were presented as Read codes [23–25]. Read codes are mapped to other nomenclatures such as the International Classification of Diseases (ICD), the International Classification of Primary Care (ICPC), and the Systematised Nomenclature for Medicine – Clinical terms (SNOMED CT) [24,25]. CPRD consists of two primary care databases: GOLD and Aurum [22]. CPRD GOLD contains data from general practices utilising the Vision software, which employs Read codes for clinical coding [26]. This study used only the CPRD GOLD database up to 2019; therefore, all variables were defined using Read codes.

### Linked data

The CPRD dataset included established linkages to Hospital Episode Statistics (HES), death registration from the Office for National Statistics, and Index of Multiple Deprivation scores [23]. Hospital Episode Statistics Admitted Patient Care (APC) is the administrative dataset for hospital admissions within the NHS in England, in addition to privately treated patients in NHS hospitals, and care delivered by treatment centres that are funded by the NHS [27]. Each HES record contains clinical information about diagnoses, patient demographic information, and administrative information such as dates and methods of admission and discharge [27]. The Office for National Statistics (ONS) provides data on causes of death [28]. The Index of Multiple Deprivation (IMD) is a geographically-based measure of socio-economic deprivation in England based on seven domains which include *‘income’, ‘employment’, ‘health deprivation and disability’,* ‘*education and skills training’, ‘crime’, ‘barriers to housing and services’,* and *‘living environment’* [29]. Higher IMD scores indicate higher levels of deprivation within the area. The IMD was represented in quintile scores based on the general practice location where patients were registered.

### Ethical approval and reporting

This study was approved by the CPRD Independent Scientific Advisory Committee (ISAC protocol number 19_050R), and access to the data was obtained on 20^th^ March 2020. It followed the Reporting of Studies Conducted Using Observational Routinely-collected Data (RECORD) guidelines [30].

### Case definitions

Patients with up-to-standard data from at least one year before their dementia diagnosis date, and born before 1954, were extracted from the CPRD GOLD database [21].

Patients with dementia were identified using the following algorithm: The earliest record of (i) a dementia diagnosis Read code or (ii) British National Formulary (BNF) and product codes for dementia-specific drug prescriptions in their record. Dementia diagnosis codes were generated from a published code list by Kontopantelis et al. [31], and the dementia drug prescription codes were BNF and product codes generated from the CPRD GOLD Code Browser Version 3.0.0 [21] (Supplementary Table S1 in S1 File). Patients coded for the dementia types Alzheimer’s disease, Vascular dementia, Mixed dementia, Dementia with Lewy Bodies and Alcohol-related dementia were identified along with their dementia diagnosis date. Patients who did not have a specific dementia type code, or had codes for more than one dementia type on or after their diagnosis date, were classed as ‘Unspecified dementia’. All patients were aged 65 years or older at the time of their dementia diagnosis. Patients with codes for early onset dementia or diagnosis prior to age 65 were excluded [32]. Dementia diagnoses or prescriptions for dementia medication could occur at any point before or after the study start date of 1^st^ April 1997, provided the diagnosis was made on or after the patient’s 65th birthday.

Eligible patients had at least one acute general hospital admission during the study period, identified in the linked HES inpatient admission data, which spanned from 1^st^ April 1997–30^th^ November 2018. The first recorded hospital admission occurring at any point after the dementia diagnosis was defined as the index hospital admission. As the outcome of interest was hospital readmission, the cohort was restricted to patients who survived the index hospital admission and were alive when discharged. Additionally, patients were required to have at least 180 days of follow-up after their index hospital admission date to ascertain hospital readmission.

Patients with a recorded hospital readmission were followed for up to one year after the readmission date, or until the first occurrence of death or transfer out of the general practice within that year. Patients without a recorded hospital readmission were followed for up to 180 days after the index hospital admission date, or until the first occurrence of death or transfer out of the general practice within that time.

### Exposures

The following determinants (risk factors) were selected based on their established clinical relevance to multimorbidity, transitional care, medication safety, and residential care context, which may influence post-hospital discharge vulnerability and subsequent hospital readmission risk among older adults with dementia.

Within routinely collected healthcare data, frailty is commonly captured using the deficit accumulation model, which conceptualises frailty as the cumulative burden of health deficits, such as diagnoses, cognitive or physical impairment, laboratory abnormalities, or malnutrition [33]. Although frailty was not directly measured as a distinct exposure, the cohort likely included individuals with varying levels of health deficits, given the high prevalence of multimorbidity and functional decline commonly observed in dementia populations [13,34].

Exposures were identified up to patients’ index hospital admission date. Code lists for all exposures are provided in Table S2 in S1 File, and the programming code used for variable development and statistical analyses is available in a GitHub repository.

### Long-term conditions

Published lists of Read codes for long-term conditions were downloaded from Opencodelist [35] and the Health Data Gateway Phenotype Library [36], mapped to the Quality Outcomes Framework (QOF) where available [37], or developed by the research team using the CPRD GOLD Code Browser [21]. Long-term conditions included diabetes mellitus (type 1 and 2), cardiovascular disease, history of stroke, cerebrovascular disease, heart failure, chronic obstructive pulmonary disease (COPD), chronic kidney disease, depression, anxiety, and insomnia. Binary variables were created, indicating whether patients had or did not have a code for the condition. All long-term conditions were chosen based on being the most common conditions that older adults with dementia live with across the UK [38–40].

### Polypharmacy

Polypharmacy is defined as the prescribing of multiple medications for one or more conditions, which can be inappropriate, and the intended benefit of the use of all medications may not be recognised [41,42]. Older adults with dementia commonly live with multimorbidity, and are likely to be discharged from hospital with multiple medications in addition to their regular medications, which may contribute to an increased prevalence of polypharmacy [43,44]. Additionally, existing literature in England and Taiwan demonstrates increased risks of multiple hospital admissions among people with dementia and polypharmacy [44,45]. Therefore, this factor was selected as an exposure to examine its association with 180-day readmission using English primary care records.

All prescribed medications were extracted from the ‘Therapy’ file in CPRD GOLD [21]. Repeat prescriptions were determined by allowing a maximum 90-day gap between each coded prescription. Prescriptions with different product codes were considered as distinct. Patients were classified as a polypharmacy case if they had at least five distinct repeat prescriptions occurring within any 90-day period between their dementia diagnosis date and index hospitalisation date [42].

### Antipsychotic medication

Previous studies have shown that older adults with dementia who experienced 30-day hospital readmissions had a higher frequency of antipsychotic prescriptions, with at least two prescriptions within one year (12.7% in patients with readmission vs 9.2% in patients with no readmission; p < 0.001) [46]. Therefore, patients with at least two prescriptions for antipsychotic medication within a 12-month period between their dementia diagnosis date and index hospitalisation date, were identified using a code list of antipsychotic medications published by Stocks et al. [47].

### Medication reviews

As recommended by NICE guidance, medication reviews in primary care should be conducted at least annually, in which the frequency of reviews should be tailored to individual need [48]. However, the most recent NHS primary care dementia data as of January 2026 shows that less than half (48%) of individuals with dementia received a medication review within the last 12 months [49]. Given that regular medication reviews may reduce the risk of adverse drug events, falls, and inappropriate prescribing [50], this factor was examined to determine its association with hospital readmission as an adverse health outcome. Patients who had at least one medication review within 12 months before their index hospitalisation date were identified using Read codes from the CPRD GOLD Code Browser Version 3.0.0 [21].

### Primary care appointments post-hospital discharge

Timely primary care follow-up after hospital discharge is considered an important component of transitional care for older adults with complex health needs [51]. Early contact with a primary healthcare professional may facilitate the monitoring of clinical deterioration and coordination of community-based support, potentially mitigating the risk of hospital readmission [52,53]. Considering the cognitive impairment and multimorbidity commonly observed among people with dementia, the presence of early post-discharge primary care consultation was examined as a determinant of 180-day readmission.

Patients who had a primary care appointment with a healthcare professional within two weeks of their index hospital discharge [54], were identified using consultation-type codes from the CPRD GOLD database [21]. Appointments were initially identified as a binary variable, and were then divided into routine and acute visits.

### Residential care

Approximately 70% of care home residents across England live with dementia [55,56]. International studies have demonstrated that older adults with dementia who live in residential care homes or nursing homes, have a reduced risk of hospital readmission [57,58]. One study conducted in London, UK, demonstrated similar results of reduced odds of 6-month readmission [59]. However, these findings were only observed from individuals with Alzheimer’s Disease and treated by mental health services within a south London NHS trust [59]. As such, the present study therefore examined the likelihood of hospital readmission of care home residents using a broader sample of primary care records of people with dementia across England. Patients coded as living in residential care prior to their index hospital admission date were classified in a binary variable. This was based on Read codes from the CPRD GOLD Code Browser, referring to patients living in a nursing home or residential care home [21]. Codes for patients living in private residences were not extracted, as this data is not routinely recorded in primary care data compared to individuals in residential care [60].

### Body mass index

The height and weight data in the ‘Additional’ files of the CPRD database were collected and supplemented by BMI Read codes to calculate BMI values. If more than one BMI value was recorded for the same patient, the most recent BMI value prior to the index hospital admission was selected. Body mass index was categorised according to the World Health Organization (WHO) criteria into underweight, healthy weight, overweight and obese [61], with ‘healthy weight’ used as the reference category in the analyses.

### Age

The age of patients on their index hospital admission date was determined by calculating the difference between the year of their index hospital admission and their year of birth. For analysis, age was then categorised into five-year intervals.

### Ethnicity

Ethnicity was determined using data from both CPRD GOLD and HES inpatient records to minimise incomplete data. Ethnicity recorded at any time in patients’ medical records was identified and categorised into the broader groups White, Black, Asian, Mixed and Other. Missing ethnicity data (4.3%) was classified as ‘Unknown’.

### Outcome measure

The primary outcome measure was all-cause 180-day hospital readmission. A 180-day period was selected given the risk that prolonged post-hospital discharge vulnerability may not be fully captured within a 30-day readmission period, and evidence demonstrating substantially increased readmission rates at 180 days compared with 30 days in dementia populations (42% vs 18%) [62]. While extending the observation period increases the possibility of capturing admissions not directly related to the index hospitalisation, this timeframe reflects sustained post-hospital discharge health instability for people with dementia, rather than immediate complications alone. In the absence of national dementia-specific readmission rates [12,63], 180-day readmission was considered an appropriate and clinically meaningful outcome measure.

180-day readmission was defined as at least two hospital admissions occurring within a maximum of 180 days apart, during the study period between 1^st^ April 1997 and 30^th^ November 2018. Inpatient hospital admissions were identified using ICD-10 codes, and obtained from HES data.

The secondary outcome measure was mortality within one year following a 180-day readmission within the study period. Mortality status was determined using death records from linked ONS data. All variable definitions and data sources of exposures and outcomes are presented in Table S3 in S1 File.

### Data cleaning

Patients with records which were missing gender, age or year of birth, GP registration date, hospital admission or discharge dates were excluded. BMI had 13.9% missing data. A complete case analysis approach was applied for all other exposures, and a sensitivity analysis was conducted to compare effect sizes between models that included variables with missing data categories and those that excluded them. Minimal differences in effect sizes were observed, and BMI was not retained in the final logistic regression models following stepwise selection (results not shown).

The code used for data preparation and analyses is available in Supplementary Materials S4 in S1 File.

### Statistical analysis

Descriptive frequencies were calculated for all sample characteristics and variables. The dichotomous outcome of a 180-day hospital readmission was defined for the cohort, and unadjusted and adjusted odds ratios (OR) with 95% confidence intervals (CI) were derived in logistic regression models. Multivariable models used backward, forward, and bidirectional stepwise selection using the Akaike Information Criterion (AIC) guiding variable selection. This analytical approach was chosen to explore and quantify associations between demographic and clinical characteristics, and the likelihood of hospital readmission among older adults living with dementia. Additionally, stepwise variable selection using the AIC was employed to support parsimonious model development and reduce the risk of model overparameterisation [64]. Two models were run; one used the count of long-term conditions (model 1) and the second entered each long-term condition as a separate entity (model 2).

As a sensitivity analysis, the post-hospital discharge primary care appointment variable was recoded using consultation Read codes into three categories including no visit, routine visit, and acute visit. Logistic regression analysis was repeated using this recoded variable in place of the original primary care appointment variable, to examine whether hospital readmission odds varied across appointment subgroups. Secondly, an interaction term between antipsychotic prescribing and the index hospital admission year group was included to assess whether the association between antipsychotic use and hospital readmission varied across the study period. Lastly, antipsychotic prescribing differed by residential status, with a higher proportion of prescriptions among patients living in residential care compared with those not living in residential care (24% vs 12%). Logistic regression models were stratified on care home status to examine if the relationship between antipsychotic prescriptions and hospital readmission differed by residential status.

A secondary outcome analysis was conducted using Cox proportional hazards regression, to identify the factors influencing one-year mortality in the subset of patients who experienced a hospital readmission. Unadjusted and adjusted hazard ratios (HR) were obtained for each exposure. Forward, backward, and bidirectional stepwise selection with AIC guiding variable selection was applied to the Cox regression model. As the values were equivalent across forward, backward, and bidirectional procedures, the bidirectional model was retained as the final model for both the logistic and Cox regression analyses.

All exposures that were retained in the logistic and Cox regression models following stepwise selection were adjusted for in the final models. Analyses were performed in RStudio version 4.4.2 [65].

## Results

### Summary

**Fig 1:**
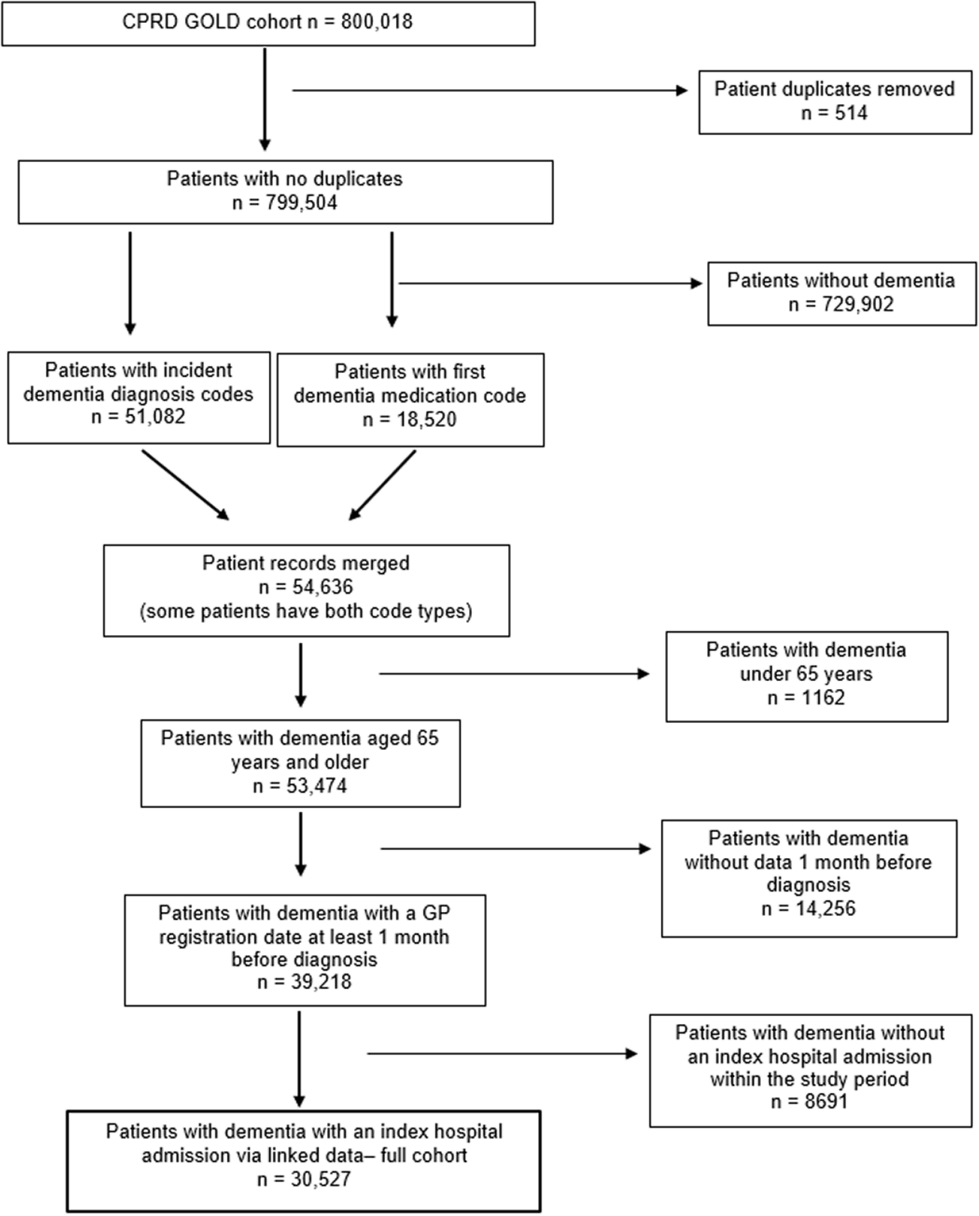
Flow chart of dementia cohort selection.

A total of 800,018 unique patients were obtained from the CPRD GOLD database. 30,527 patients registered at 253 general practices in England met the eligibility criteria. The process of identifying the final dementia cohort is outlined in Fig 1.

### Descriptive characteristics

The mean age at index hospitalisation was 83.6 years (SD 6.7), with a median of 84 years (IQR 79.0–88.0). 47.5% of patients were aged 85 and over, and 62.7% were female (vs 37.3% male). Ethnicity data showed that 93.4% of patients were White, with smaller proportions for all other ethnic groups (0.9% Asian, 0.7% Black, 0.2% Mixed, 0.5% Other, 4.3% Unknown). Additionally, general practices were based in slightly more deprived areas of England, with 27.3% of patients registered at general practices recorded to have an IMD score of 5. Only 4.3% of patients were recorded to live in residential care before their index hospitalisation. Descriptive data for the dementia cohort are outlined in Table 1.

**Table 1:** Demographic characteristics of total cohort.

	Dementia cohort N	%	Readmitted patients N	%	Non-readmitted patients N	%	p-value^†^
Total number of patients	30,527	100	12,351	100	18,176	100	
Years to index admission							<0.001
Mean (SD) Median (IQR) Range	1.4 (1.7) 1.0 (0.0-2.0) 0.0-17.0		1.2 (1.5) 1.0 (0.0-2.0) 0.0-17.0		1.5 (1.8) 1.0 (0.0-2.0) 0.00-16.0		
Days to readmission							
Mean (SD) Median (IQR) Range	N/A		59.2 (49.5) 45.0 (17.0-94.0) 0.0-179.0		N/A		–
Age at index hospital admission (continuous)							<0.001
Mean (SD) Median (IQR) Range	83.6 (6.7) 84.0 (79.0-88.0) 65.0-105.0		83.5 (6.6) 84.0 (79.0-88.0) 65.0-104.0		83.8 (6.8) 84.0 (79.0-89.0) 65.0-105.0		
Age at index hospital admission (categorical)							<0.001
65-69 70-74 75-79 80-84 85-89 90+	728 2305 5057 7946 8504 5987	2.4 7.6 16.6 26.0 27.9 19.6	296 937 2068 3283 3512 2255	2.4 7.6 16.7 26.6 28.4 18.3	432 1368 2989 4663 4992 3732	2.4 7.5 16.4 25.7 27.5 20.5	
Gender (readmission rate)							<0.001
Male Female	11,373 19,154	37.3 62.7	4940 7411	40.0 (43.4) 60.0 (38.7)	6433 11,743	35.4 64.6	
Ethnicity							<0.001
White Asian Black Mixed Other Unknown	28,505 263 218 51 163 1327	93.4 0.9 0.7 0.2 0.5 4.3	11,748 107 97 19 48 332	95.1 0.9 0.8 0.1 0.4 2.7	16,757 156 121 32 115 995	92.2 0.9 0.7 0.2 0.6 5.4	
Region							<0.001
North East North West Yorkshire & The Humber East Midlands West Midlands East of England South West South Central London South East Coast	1110 3587 1852 1279 2760 3866 5633 3920 2308 4212	3.6 11.8 6.1 4.2 9.0 12.7 18.5 12.8 7.6 13.8	465 1438 768 533 1155 1559 2367 1355 1011 1700	3.7 11.6 6.2 4.3 9.4 12.6 19.2 11.0 8.2 13.8	645 2149 1084 746 1605 2307 3266 2565 1297 2512	3.5 11.8 6.0 4.1 8.8 12.7 18.0 14.1 7.1 13.9	
GP IMD quintile							<0.001
1 (least deprived) 2 3 4 5 (most deprived)	4350 4443 5763 7627 8344	14.2 14.6 18.9 25.0 27.3	1763 1638 2407 3127 3416	14.3 13.3 19.5 25.3 27.7	2587 2805 3356 4500 4928	14.2 15.4 18.5 24.8 27.1	
Living in residential care	1308	4.3	410	3.3	898	4.9	<0.001

GP IMD = General practice surgery index of multiple deprivation.

† p-values from Mann Whitney U test (continuous) and Chi-square test (categorical).

### Clinical characteristics

The number of recorded dementia diagnoses within the cohort increased between 1997 and 2012, rising from 453 in 1997–1998–4,700 in 2011–2012. After this period, the number of diagnoses declined to 934 by 2017–2018, which may reflect changes in general practice participation with the CPRD GOLD database over time. Alzheimer’s disease was the most documented dementia subtype in patients’ primary healthcare records, accounting for 30.8% of cases, followed by vascular dementia at 25.2%. Less frequently recorded subtypes included dementia with Lewy bodies, mixed dementia, and alcohol-related dementia. The largest group of patients (41.7%) did not have a specific dementia subtype recorded at the time of diagnosis. The average time from dementia diagnosis to index hospital admission was 1.4 years (SD 1.7), with a median of one year (IQR 0.0–2.0). Most of the cohort died during the study period (82.2%). These findings are further detailed in Table 2.

**Table 2:** Clinical characteristics of total cohort.

	Dementia cohort N	%	Readmitted patients N	%	Non-readmitted patients N	%	p-value†
Total number of patients	30,527	100	12,351	100	18,176	100	
Dementia type							<0.001
Unspecified Alcoholic Alzheimer’s DLB Mixed VaD	12,720 67 9412 456 182 7690	41.7 0.2 30.8 1.5 0.6 25.2	5120 28 3657 192 82 3272	41.5 0.2 29.6 1.6 0.7 26.5	7600 39 5755 264 100 4418	41.8 0.2 31.7 1.5 0.6 24.3	
BMI							0.03
Underweight Healthy weight Overweight Obese Missing	2036 12,555 8165 3589 4182	6.7 41.1 26.7 11.8 13.7	768 5114 3411 1520 1538	6.2 41.4 27.6 12.3 12.5	1268 7441 4754 2069 2644	7.0 40.9 26.2 11.4 14.5	
COPD	2393	7.8	1120	9.1	1273	7.0	<0.001
Cardiovascular Disease	7264	23.8	3214	26.0	4050	22.3	<0.001
Heart Failure	2600	8.5	1164	9.4	1436	7.9	<0.001
Chronic Kidney Disease	7272	23.8	3160	25.6	4112	22.6	<0.001
Type 1 Diabetes	322	1.1	161	1.3	161	0.9	<0.001
Type 2 Diabetes	4550	14.9	2075	16.8	2475	13.6	<0.001
Cerebrovascular Disease	2545	8.3	1097	8.9	1448	8.0	0.005
History of stroke	1584	5.2	711	5.8	873	4.8	<0.001
Depression	6750	22.1	2873	23.3	3877	21.3	<0.001
Anxiety	4998	16.4	2070	16.8	2928	16.1	0.13
Insomnia	4463	14.6	1844	14.9	2619	14.4	0.21
Patients per LTC category							<0.001
0 1 2-3 4-5 6+	8516 9115 10,399 2281 216	27.9 29.9 34.1 7.5 0.7	3104 3584 4472 1075 116	25.1 29.0 36.2 8.7 0.9	5412 5531 5927 1206 100	29.8 30.4 32.6 6.6 0.6	
Polypharmacy	23,397	76.6	9634	78.0	13,763	75.7	<0.001
Antipsychotic medication	3834	12.6	1435	11.6	2399	13.2	<0.001
Medication reviews	13,333	43.7	5655	45.8	7678	42.2	<0.001
Primary care appointment within 2 weeks of index discharge	13,277	43.5	5773	46.7	7504	41.3	<0.001
Patients who died within study period	25,087	82.2	10,357	83.9	14,730	81.0	–

DLB = Dementia with Lewy Bodies, VaD = Vascular dementia, BMI = Body Mass Index, COPD = Chronic Obstructive Pulmonary Disease, LTC = Long-term condition.

† p-values from Mann Whitney U test (continuous) and Chi-square test (categorical).

Multimorbidity was prevalent within the cohort, with 34.1% of patients diagnosed with two to three long-term conditions. The most recorded conditions were chronic kidney disease (23.8%), cardiovascular disease (23.8%), and depression (22.1%). Regarding BMI, 41.4% of patients were classified as having a healthy weight, while 26.7% were overweight, 11.8% were obese and 6.7% were underweight. Polypharmacy was highly prevalent, with 76.6% of patients prescribed five or more medications within a 90-day period. Among patients classified as having polypharmacy, 89.5% met this definition within the year before their index hospital admission date. At least two antipsychotic medications were prescribed to 12.6% of the cohort within 12 months at any time between their dementia diagnosis date and their index hospitalisation date. Among these patients, 86.5% were observed to have at least two antipsychotic medication prescriptions within the year before their index hospital admission date. 43.7% of patients had undergone a medication review within 12 months before their index hospitalisation, and 43.5% of patients had a primary care consultation within two weeks of their index hospital discharge (Table 2).

### Patients with 180-day readmission

Among the 30,527 patients with dementia who had at least one hospital admission, 12,351 (40.5%) experienced a hospital readmission within 180 days. The mean time to readmission was 59.2 days (SD 49.5), with a median of 45 days (IQR 17.0–94.0). Readmission rates differed by gender, with male patients showing a higher rate (43.4%) compared to female patients (38.7%). The mean age of readmitted patients was 83.5 years (SD 6.6), with the largest age group (28.4%) aged between 85 and 89 years. Additionally, 3546 patients (28.7%) were observed to have a recorded death within one year of their hospital readmission date. Among these patients, the mean time to death was 228.6 days (≈7.5 months; SD 142.6), with a median time of 278 days (IQR 77–366). Further details on the characteristics of readmitted patients are presented in Table 2.

### Associations between predictors and 180-day hospital readmission

#### Univariate logistic regression.

Univariate logistic regression was performed to assess the association between individual predictor variables and the likelihood of a 180-day readmission. The odds ratio (OR), 95% confidence intervals (CI), and p-values are reported in Table 3. Variables which showed positive univariate associations with the outcome were being male, having one or more long term conditions (with odds increasing with more conditions), increase in GP IMD quintile, and polypharmacy. The long-term conditions most associated with readmission were COPD, type 1 and type 2 diabetes, and cardiovascular disease. Having Alzheimer’s disease (compared to unspecified dementia), being prescribed antipsychotic medication, and living in residential care were associated with reduced odds of readmission.

**Table 3:** Association between patient exposures and 180-day hospital readmission.

Variable (reference category)	Unadjusted OR (95% CI)	Model 1: adjusted OR^‡^ (95%CI)	Model 1: p-value (multivariate)	Model 2: adjusted OR^‡^ (95%CI)	Model 2: p-value (multivariate)	Note
**Gender** (Male)						
Female	0.82 (0.78-0.86)	0.84 (0.80-0.88)	<0.001*	0.85 (0.81-0.90)	<0.001*	
**Age** (65–69)						
70-74 75-79 80-84 85-89 90+	1.00 (0.84-1.19) 1.01 (0.86-1.18) 1.03 (0.88-1.20) 1.03 (0.88-1.20) 0.88 (0.75-1.03)	1.03 (0.86-1.22) 1.04 (0.89-1.23) 1.08 (0.92-1.26) 1.09 (0.94-1.28) 0.97 (0.83-1.14)	0.77 0.59 0.34 0.26 0.75	1.02 (0.86-1.21) 1.04 (0.88-1.22) 1.07 (0.92-1.25) 1.09 (0.93-1.27) 0.98 (0.83-1.15)	0.82 0.65 0.38 0.29 0.77	
**Ethnicity** (White)						
Asian Black Mixed Other Unknown	0.98 (0.76-1.25) 1.14 (0.87-1.49) 0.85 (0.47-1.48) 0.60 (0.42-0.83) 0.48 (0.41-0.54)	0.89 (0.70-1.15) 1.08 (0.83-1.42) 0.83 (0.46-1.45) 0.60 (0.42-0.84) 0.51 (0.45-0.58)	0.38 0.56 0.51 0.003* <0.001*	0.87 (0.68-1.11) 1.06 (0.80-1.38) 0.81 (0.45-1.42) 0.60 (0.43-0.84) 0.51 (0.45-0.58)	0.27 0.69 0.47 0.004* <0.001*	
**BMI** (Healthy weight)						
Underweight Overweight Obese Missing	0.88 (0.80-0.97) 1.04 (0.99-1.10) 1.07 (0.99-1.15) 0.85 (0.79-0.91)	–	–	–	–	Not included in multivariate models⁺
**Type** (Unspecified)						
Alcoholic Alzheimer’s DLB Mixed VaD	1.07 (0.65- 1.73) 0.94 (0.89-1.00) 1.08 (0.89-1.30) 1.22 (0.91-1.63) 1.10 (1.04-1.16)	1.05 (0.63-1.70) 0.93 (0.88-0.98) 1.01 (0.84-1.23) 1.13 (0.84-1.52) 1.00 (0.94-1.06)	0.86 0.006* 0.89 0.41 0.97	–	–	Not included in multivariate model 2⁺
**GP IMD**	1.02 (1.00-1.04)	1.01 (1.00-1.03)	0.11	1.01 (1.00-1.03)	0.16	
**LTC category**						
1 2-3 4-5 6+	1.13 (1.06-1.20) 1.32 (1.24-1.40) 1.55 (1.42-1.71) 2.02 (1.54-2.66)	1.11 (1.04-1.18) 1.26 (1.19-1.34) 1.46 (1.33-1.60) 1.85 (1.40-2.43)	0.001* <0.001* <0.001* <0.001*			Not included in multivariate model 2⁺
**COPD**	1.32 (1.22-1.44)	–	–	1.23 (1.13-1.34)	<0.001*	
**Cardiovascular Disease**	1.23 (1.16-1.29)	–	–	1.12 (1.06-1.18)	<0.001*	
**Heart failure**	1.21 (1.12-1.32)	–	–	1.11 (1.02-1.21)	0.01*	
**Chronic Kidney Disease**	1.18 (1.11-1.24)	–	–	1.09 (1.03-1.15)	0.003*	
**Type 1 Diabetes**	1.48 (1.19-1.84)	–	–	1.25 (1.00-1.57)	0.05*	
**Type 2 Diabetes**	1.28 (1.20-1.36)	–	–	1.17 (1.10-1.25)	<0.001*	
**Cerebrovascular Disease**	1.13 (1.04-1.22)	–	–	1.07 (0.98-1.16)	0.14	
**Stroke history**	1.21 (1.09-1.34)	–	–	1.10 (0.99-1.22)	0.07	
**Depression**	1.12 (1.06-1.18)	–	–	1.12 (1.06-1.18)	<0.001*	
**Anxiety**	1.05 (0.99-1.12)	–	–	–	–	Not included in multivariate models⁺
**Insomnia**	1.04 (0.98-1.11)	–	–	–	–	Not included in multivariate models⁺
**Primary care appointment after discharge**	1.25 (1.19-1.31)	1.21 (1.15-1.27)	<0.001*	1.21 (1.15-1.27)	<0.001*	
**Antipsychotic medication**	0.86 (0.81-0.93)	0.90 (0.84-0.96)	0.003*	0.91 (0.85-0.98)	0.01*	
**Polypharmacy**	1.14 (1.08-1.20)	–	–	–	–	Not included in multivariate models⁺
**Medication reviews**	1.15 (1.10-1.21)	1.09 (1.04-1.14)	<0.001*	1.09 (1.04-1.14)	<0.001*	
**Residential care**	0.66 (0.59-0.74)	0.66 (0.59-0.75)	<0.001*	0.66 (0.59-0.75)	<0.001*	

‡ All ORs are adjusted for the other predictors retained after AIC bidirectional stepwise selection.

⁺Not selected for multivariate analysis via AIC bidirectional stepwise selection.

*Significant p-value set at p < 0.05.

BMI = Body Mass Index, LTC = Long-term condition, DLB = Dementia with Lewy Bodies, VaD = Vascular dementia.

#### Multivariate logistic regression.

Multivariate logistic regression models are summarised in Table 3. Backward, forward, and bidirectional stepwise selection resulted in the removal of BMI, anxiety, insomnia and polypharmacy from the initial model, while all other exposures were retained. Factors with a significant effect within the model (increased odds of readmission) included being male, having more long-term conditions, having a medication review within a year of hospitalisation, or attending a primary care appointment post-discharge. Having Alzheimer’s, ‘Other’ ethnicity, prescriptions for antipsychotic medication, and living in residential care, were associated with lower odds of readmission in the adjusted model. However, the area under the curve (AUC = 0.57) for this adjusted model indicated limited ability in distinguishing between readmitted and non-readmitted patients. Although the analysis was explanatory rather than predictive, the AUC is reported to provide transparency regarding model discriminatory performance.

In the second adjusted logistic regression model, which included individual long-term conditions, COPD, type 1 and type 2 diabetes, and cardiovascular disease showed the highest odds of readmission. History of stroke showed tendency towards increased odds but did not reach statistical significance. The odds of readmission and significance levels of all other variables remained unchanged from the first adjusted logistic regression model. The area under the curve (AUC = 0.57) for this model also indicated limited discriminatory ability in distinguishing between readmitted and non-readmitted patients.

In the sensitivity analyses, it was found that compared with no primary care visit, both routine visits (OR = 1.20, p < 0.001) and acute visits (OR = 1.53, p < 0.001) were associated with increased odds of hospital readmission. The interaction term between antipsychotic prescribing and hospital admission year group did not significantly improve the fit in either model (likelihood ratio test p = 0.34). Stratified logistic regression analyses showed similar associations between antipsychotic prescribing and hospital readmission in both groups (residential care OR = 0.95, p = 0.75; non-residential care OR = 0.91, p = 0.01) (results presented in Supplementary Tables S5-S7 in S1 File). These findings suggest no evidence that the relationship between antipsychotic prescriptions and hospital readmission differed by residential status or year of admission.

#### Mortality risk after 180-day readmission.

Results of the Cox proportional hazards regression model are shown in Table 4. The model showed a significant effect on increasing mortality risk with age, being male, being underweight, having two or more long-term conditions, polypharmacy, and living in residential care. Notably, patients prescribed at least two antipsychotic medications within any 12-month period between their dementia diagnosis and index hospitalisation, demonstrated a 35% increased risk of mortality after hospital readmission.

**Table 4:** Mortality risk within one year of hospital readmission.

Variable (reference category)	Adjusted HR ^‡^ (95%CI)	p-value
**Gender** (Male)		
Female	0.67 (0.63-0.72)	<0.001*
**Age** (65–69)		
70-74	1.14 (0.84-1.53)	0.40
75-79	1.39 (1.05-1.83)	0.02*
80-84	1.72 (1.31-2.26)	<0.001*
85-89	2.21 (1.68-2.89)	<0.001*
90+	2.90 (2.21-3.81)	<0.001*
**Ethnicity** (White)		
Asian	0.63 (0.41-0.98)	0.04*
Black	0.88 (0.58-1.34)	0.56
Mixed	0.49 (0.12-1.98)	0.32
Other	0.82 (0.47-1.45)	0.50
Unknown	1.85 (1.57-2.19)	<0.001*
**Type** (Unspecified)		
Alcoholic	1.64 (0.88-3.06)	0.12
Alzheimer’s	0.89 (0.82-0.97)	0.006*
DLB	1.19 (0.93-1.52)	0.16
Mixed	1.11 (0.76-1.62)	0.60
VaD	1.02 (0.94-1.11)	0.64
**BMI** (Healthy weight)		
Underweight	1.33 (1.16-1.52)	<0.001*
Overweight	0.83 (0.76-0.90)	<0.001*
Obese	0.80 (0.71-0.90)	<0.001*
Missing	1.12 (1.02-1.24)	0.02*
**LTC category**		
1	1.00 (0.91-1.10)	0.94
2-3	1.18 (1.08-1.29)	<0.001*
4-5	1.32 (1.16-1.50)	<0.001*
6+	1.60 (1.19-2.15)	0.002*
**Polypharmacy**	1.14 (1.05-1.25)	0.003*
**Antipsychotic medication**	1.35 (1.23-1.49)	<0.001*
**Residential care**	1.20 (1.02-1.41)	0.03*

Hazard ratios correspond to the final multivariable Cox model selected using bidirectional stepwise selection.

‡ All HRs are adjusted for the other predictors shown in the table.

*Significant p-value set at p < 0.05.

DLB = Dementia with Lewy Bodies, VaD = Vascular dementia, LTC = Long-term condition.

## Discussion

### Summary

This study highlighted that older adults living with dementia who also had multiple long-term conditions, received a medication review within a year before their index hospital admission, or had a primary care appointment after discharge, had significantly greater odds of experiencing a 180-day hospital readmission. Among individual long-term conditions, COPD was associated with the highest odds of readmission. The likelihood of readmission was significantly lower among female patients, individuals identifying as ‘Other’ ethnicity, and those diagnosed with Alzheimer’s disease compared to individuals with an unspecified dementia diagnosis. Living in residential care was associated with a reduced risk of hospital readmission of up to 34% across adjusted multivariate models.

Among patients who experienced a hospital readmission, Cox proportional hazards regression showed a direct association between age and one-year mortality risk. Mortality risk also increased with multiple long-term conditions and polypharmacy. While exposure to at least two antipsychotic medications before index hospitalisation was associated with reduced odds of readmission, it was linked to a higher mortality risk among those who were readmitted. Notably, underweight patients had a 33% increased mortality risk, while overweight and obese patients had reduced mortality risks following readmission. Furthermore, patients living in residential care had a 20% increased mortality risk within one year of readmission. Female patients and those diagnosed with Alzheimer’s disease continued to have a lower mortality risk after readmission.

### Interpretation of results

The findings of this cohort study suggest that biological, psychological, and social factors interact to influence hospital readmissions in older adults with dementia. While this study does not establish a causal relationship between specific determinants and hospital readmission, the consistency of findings across this and prior research suggests that findings are robust across different settings [46,4,66–68]. Cummings et al. [66] and Rudolph et al. [67] both found that female patients with dementia had a lower likelihood of hospital readmission compared to male patients. Additionally, Rudolph et al. [67] identified an increased risk of readmission with age.

### Long-term conditions

Previous studies have also shown a strong association between long-term conditions and hospital readmission [4,67,68]. Despite differences in the measurement of comorbidity, where Rudolph et al. [67] applied the Charlson Comorbidity Index, while this study investigated individual conditions, both studies found comorbidity to be the strongest determinant of hospital readmission. Our study identified COPD, cardiovascular disease, diabetes and depression as key conditions associated with hospital readmission. This is consistent with previous studies, which reported strong associations between readmission and cardiovascular disease [4], type 2 diabetes and heart failure [68].

### Psychosocial determinants

Most importantly, this study found contrasting results regarding prescribed antipsychotic medication. It is well known that mortality risk is consistently elevated in dementia patients prescribed antipsychotics, which led to UK prescribing policy changes [69,70], and our study is consistent with this. However, our study is in contradiction to other research showing hospital admissions are increased in antipsychotic users with dementia, particularly current users, or those taking a higher dosage, with the main mechanism being via injury, e.g., falls [71]. Although for readmission, the antipsychotic-specific contribution remains underexplored in the primary literature, having been explored only in an American study [46] and showing a positive association. Our study observed a negative association between antipsychotic use and readmission, although antipsychotic usage was positively associated with mortality. This may be due to some level of confounding, e.g., collider bias or confounding by indication, or some level of difference on discharge such as increased care (there were higher levels of antipsychotic prescribing in care-home residents for example) or monitoring, rather than representing a causal protective effect. Furthermore, the extended study period between 1997–2018 covers substantial changes in dementia management and clinical guidelines [40,72]. Pooling data across this timeframe may obscure the changing prescribing practices of antipsychotic medications and patient characteristics that are specific to particular periods.

Recent studies have similarly reported a reduced likelihood of hospital readmission among those living in residential care. A cohort study in Denmark found that older care home residents with dementia were less likely to experience a 30-day hospital readmission [58]. Similarly, the service evaluation of an advance care planning education program for care home staff in Lincolnshire, UK, led to a 55% reduction in recurrent hospital admissions for residents with dementia over a three-year period [73]. These findings may reflect the continuous care provided in residential settings, where the management of older residents with dementia prioritises the prevention of hospitalisations [58,73].

### Opportunities for clinical intervention to prevent readmission

While other studies have found that medication reviews performed by clinical pharmacists significantly reduce the risk of medication-related hospital readmissions in older adults, including those with dementia [74], our findings revealed a slightly increased risk of readmission in patients who had a recent medication review. Therefore, it is not clear that these reviews work well to reduce admissions, although they may still reduce medication-related adverse events. The finding that there was an increased risk of readmission for patients who had a primary care appointment after their index discharge was also unexpected, as the assumption would be that appropriate primary or community care would help to prevent hospitalisations. Instead, this likely represents confounding by indication, where primary care contact may function less as an intervention to improve or maintain health out of the hospital, and more as a marker of unresolved care needs or clinical vulnerability during a critical recovery period. This is consistent with literature suggesting that such patterns of healthcare utilisation may reflect underlying vulnerability, rather than effective prevention [75,76]. Where community care interventions may not be received in a timely way, primary care may be the only part of the healthcare system that can be accessed if care needs are escalating, such that early primary care contact may be framed as a marker of a fragmented care pathway.

### Mortality

Just under one third of patients with a 180-day readmission died within the year and had an average time to death of approximately 7.5 months. This finding aligns with the cohort study by Yorganci et al. [77], which examined patterns of unplanned hospital admissions in older adults with dementia, reporting that 37.3% of all unplanned admissions occurred in the last year of life. Although Yorganci et al. [77] did not specifically investigate readmissions, both studies highlight the need to integrate advance care planning including preferences for end-of-life care settings within dementia care pathways, to improve patient outcomes and reduce multiple hospitalisations. Unplanned admissions may serve as key clinical markers for identifying individuals in need of advance care planning. This emphasises the need for a needs-based, rather than prognosis-based, approach to palliative care in dementia [78].

### Strengths and weaknesses

This study is one of the largest to investigate the association between clinical factors and 180-day hospital readmission among older adults with dementia in England. This ensures that the results are relevant to the NHS and can provide insights into improving care delivery and reducing hospital readmissions in older adults with dementia. As the data are routinely collected, this study includes data from underrepresented groups in research, such as clinically vulnerable patients, those living in deprived areas or those with low levels of health literacy [79]. Case ascertainment was strengthened by combining resources to define the exposures, confounders and outcomes of interest, by using primary care, HES, and IMD data. Additionally, this study was able to identify patients who died during the study period using death records from the ONS, which reinforced the quality of the death indicator and examined the mortality risk of readmitted patients.

Although this study had key strengths, several limitations should be acknowledged. Classifying dementia cases was particularly challenging for patients with multiple dementia types during the study period. Some patients had multiple dementia types coded on their diagnosis date, while others were initially classified as ‘Unspecified’, and later assigned a specific dementia type at subsequent appointments. As a result, these individuals were not consistently categorised under a specific dementia type, and many patients remained coded as ‘Unspecified’ in their healthcare records. This could reflect the fact that dementia subtypes were not routinely diagnosed in the UK until the early 2000s, which is reflective of the increased number of diagnoses within this decade [80,81]. Most of the study cohort were of White ethnicity, meaning that the results may not be generalised across patients of ethnic minority backgrounds, and findings related to ethnicity may not be robust. Our study spanned 1997–2018, which allowed a large sample, however clinical practice, particularly regarding antipsychotic prescribing in dementia, changed significantly during this period. While the macro-structure of care in the NHS stayed stable (GP as the primary ongoing relationship and coordinator; hospital as the destination for acute deterioration, crisis, and end of life) there were several changes, such as diagnosis and care for dementia becoming incentivised in general practice (via QOF) which resulted in overall earlier diagnosis, as well as care planning and annual reviews. Later entrants into our cohort may be systematically different from early entrants: potentially less severe at diagnosis, younger, or more likely to have been actively sought out. Coding and documentation both in GP and hospital are likely to have improved over the time period, which may change the observed associations. Effect sizes may be attenuated if variables were under-ascertained in earlier years. However, a sensitivity analysis including a sub-cohort of patients diagnosed with dementia from 2009 onwards showed no statistical significance with hospital readmission (see Supplementary Table S8 in S1 File). A further limitation is that care drivers for dementia may have substantially changed in the period spanning and subsequent to Covid-19, and therefore an updated analysis in very recent data, for comparison, is warranted. Additionally, as patients who died during their index hospital admission were excluded from the cohort, the findings may be subject to survivorship bias, which could have influenced the observed magnitude of associations between the examined determinants and the odds of hospital readmission.

Some additional variables of interest could be adopted in future work. Our study did not investigate acute infection or illness at the time of the participants’ index hospital admission, which could have provided further insight into readmission risk. Overall, the exploration of the reason for initial hospitalisation could be further developed, particularly by including trauma, such as falls and fractures, which are highly related to complex clinical sequelae. Relevant clinical biomarkers that could be included are elevated glycosylated haemoglobin (HbA1c) levels as an indicator of uncontrolled diabetes, C-reactive protein (CRP) and interleukins as markers of systemic inflammation, lactate levels suggestive of sepsis, and reduced serum sodium levels as a marker of mortality [82–85]. Future research should consider these and other biomarkers to enhance the assessment of clinical factors influencing hospital readmission. Additionally, information on over-the-counter medication use and medication adherence was not available within the dataset, both of which may have influenced the risk of readmission. More information on household composition and whether individuals have a designated family caregiver would be informative in future studies [23]. Lastly, this data only covered patients up to 2019, and following the Covid-19 pandemic, clinical services may have been reconfigured, and the health needs of people living with dementia could have increased.

## Conclusion

This study identified clinical determinants significantly associated with 180-day hospital readmission among older adults living with dementia. Multiple long-term conditions emerged as the strongest determining factor, with COPD, diabetes, cardiovascular disease, and depression showing the highest association with readmission. While living in residential care reduced the odds of hospital readmission, it was subsequently associated with a higher risk of one-year mortality after readmission. This increased mortality risk may indicate that patients who have moved to residential care have advanced dementia or are transitioning toward the end of life. Given the influence of age and multiple long-term conditions on mortality risk, future research should explore the role of frailty, as defined by cumulative deficits, and its interaction with other known determinants of hospital readmission among older adults with dementia. Future research should assess psychosocial factors such as social support, caregiver burden, and mental wellbeing, which may influence hospital discharge outcomes and readmission risk. Overall, these findings have important implications for healthcare leaders across the UK, and aligned with UK government strategy, suggest there is a need for strengthening preventive and community-based care to reduce hospital readmissions, for example, by developing targeted interventions and providing better out-of-hospital support for individuals living with dementia.

## Supporting information

S1 File Supplementary Materials File 1 (DOCX)
