# Supplementary material for: Identifying the determinants of hospital readmission in dementia patients – A retrospective cohort study using electronic healthcare records in England
Source: PLoS One. 2026 Jul 15;21(7):e0351331. doi: 10.1371/journal.pone.0351331 (PMC13372114; doi:10.1371/journal.pone.0351331)
Supplement: S1 File — (DOCX) [file pone.0351331.s001.docx]

Supplementary Materials

Contents:

Table S1 Read code lists of dementia diagnoses and covariates p01

Table S2 Read code lists of exposures p10

Table S3 All Variable Definitions and Source Data p42

S4: Data preparation and analyses code p47

Table S5: Sensitivity analysis examining primary care visit type

and hospital readmission p47

Table S6: Interaction analysis between antipsychotic prescribing

and hospital admission year group p47

Table S7: Stratified logistic regression analyses examining the association between antipsychotic prescribing and hospital readmission by residential status p48

Table S8: Association between antipsychotic medication prescribing and hospital readmission among patients diagnosed with dementia across the full study

period (1997–2018) and after 2009 p48

# Table S1: Read code lists of dementia diagnoses and covariates

## Dementia diagnosis codes

| **Readcode** | **Medcode** | **Description** |
| --- | --- | --- |
| F110.00 | 1917 | Alzheimer's disease |
| Eu00.00 | 7664 | [X]Dementia in Alzheimer's disease |
| Eu00z11 | 8195 | [X]Alzheimer's dementia unspec |
| Eu00112 | 11379 | [X]Senile dementia, Alzheimer's type |
| F110000 | 16797 | Alzheimer's disease with early onset |
| Eu00z00 | 29386 | [X]Dementia in Alzheimer's disease, unspecified |
| Eu00200 | 30706 | [X]Dementia in Alzheimer's dis, atypical or mixed type |
| F110100 | 32057 | Alzheimer's disease with late onset |
| Eu00100 | 38678 | [X]Dementia in Alzheimer's disease with late onset |
| Eu00113 | 43346 | [X]Primary degen dementia of Alzheimer's type, senile onset |
| Eu00111 | 46762 | [X]Alzheimer's disease type 1 |
| Eu01111 | 55838 | [X]Predominantly cortical dementia |
| Fyu3000 | 59122 | [X]Other Alzheimer's disease |
| Eu00013 | 61528 | [X]Alzheimer's disease type 2 |
| Eu01.00 | 6578 | [X]Vascular dementia |
| E004.11 | 8634 | Multi infarct dementia |
| Eu01200 | 8934 | [X]Subcortical vascular dementia |
| Eu01.11 | 9565 | [X]Arteriosclerotic dementia |
| Eu01100 | 11175 | [X]Multi-infarct dementia |
| Eu01z00 | 19393 | [X]Vascular dementia, unspecified |
| E004.00 | 19477 | Arteriosclerotic dementia |
| E004z00 | 42279 | Arteriosclerotic dementia NOS |
| E004000 | 43089 | Uncomplicated arteriosclerotic dementia |
| E004300 | 43292 | Arteriosclerotic dementia with depression |
| Eu01000 | 46488 | [X]Vascular dementia of acute onset |
| Eu01y00 | 55313 | [X]Other vascular dementia |
| E004200 | 55467 | Arteriosclerotic dementia with paranoia |
| E004100 | 56912 | Arteriosclerotic dementia with delirium |
| F116.00 | 7572 | Lewy body disease |
| Eu02500 | 26270 | [X]Lewy body dementia |
| Eu01300 | 31016 | [X]Mixed cortical and subcortical vascular dementia |
| Eu10711 | 26323 | [X]Alcoholic dementia NOS |
| E012.11 | 27342 | Alcoholic dementia NOS |
| E00..11 | 1916 | Senile dementia |
| Eu02z14 | 4357 | [X] Senile dementia NOS |
| Eu02z00 | 4693 | [X] Unspecified dementia |
| E000.00 | 7323 | Uncomplicated senile dementia |
| E002000 | 18386 | Senile dementia with paranoia |
| E002100 | 21887 | Senile dementia with depression |
| Eu02z16 | 27759 | [X] Senile dementia, depressed or paranoid type |
| Eu02z13 | 34944 | [X] Primary degenerative dementia NOS |
| E003.00 | 37015 | Senile dementia with delirium |
| E002z00 | 41089 | Senile dementia with depressive or paranoid features NOS |
| E002.00 | 44674 | Senile dementia with depressive or paranoid features |
| Eu02y00 | 64267 | [X]Dementia in other specified diseases classif elsewhere |

Source: Kontopantelis et al (1)

## Dementia medication codes

| **Prod code** | **BNF code** | **Product name** | **Drug substance name** |
| --- | --- | --- | --- |
| 9966 | 04110000 | Ebixa 5mg/0.5ml pump actuation oral solution (Lundbeck Ltd) | Memantine hydrochloride |
| 72344 | 04110000 | Lotprosin XL 16mg capsules (Accord Healthcare Ltd) | Galantamine hydrobromide |
| 7329 | 04110000 | Galantamine 20mg/5ml oral solution sugar free | Galantamine hydrobromide |
| 39240 | 04110000 | Memantine 20mg tablets | Memantine hydrochloride |
| 36976 | 04110000 | Rivastigmine 4.6mg/24hours transdermal patches | Rivastigmine |
| 56771 | 04110000 | Rivastigmine 3mg capsules (Dr Reddy's Laboratories (UK) Ltd) | Rivastigmine hydrogen tartrate |
| 11751 | 04110000 | Rivastigmine 3mg capsules | Rivastigmine hydrogen tartrate |
| 80554 | 04110000 | Memantine 5mg tablets | Memantine hydrochloride |
| 68845 | 04110000 | Memantine 10mg/ml oral solution sugar free (A A H Pharmaceuticals Ltd) | Memantine hydrochloride |
| 5334 | 04110000 | Reminyl 12mg tablets (Shire Pharmaceuticals Ltd) | Galantamine hydrobromide |
| 77319 | 04110000 | Zeebral XL 16mg capsules (Teva UK Ltd) | Galantamine hydrobromide |
| 53922 | 04110000 | Donepezil 10mg orodispersible tablets (Consilient Health Ltd) | Donepezil hydrochloride |
| 78024 | 04110000 | Galzemic 4mg/ml oral solution (Zentiva Pharma UK Ltd) | Galantamine hydrobromide |
| 48443 | 04110000 | Donepezil 10mg orodispersible tablets | Donepezil hydrochloride |
| 61920 | 04110000 | Luventa XL 8mg capsules (Fontus Health Ltd) | Galantamine hydrobromide |
| 11546 | 04110000 | Exelon 1.5mg capsules (Novartis Pharmaceuticals UK Ltd) | Rivastigmine hydrogen tartrate |
| 10255 | 04110000 | Galantamine 8mg modified-release capsules | Galantamine hydrobromide |
| 38976 | 04110000 | Memantine 5mg+10mg+15mg+20mg Tablet | Memantine Hydrochloride |
| 5616 | 04110000 | Exelon 6mg capsules (Novartis Pharmaceuticals UK Ltd) | Rivastigmine hydrogen tartrate |
| 68792 | 04110000 | Memantine 10mg/ml oral solution sugar free (Healthcare Pharma Ltd) | Memantine hydrochloride |
| 61385 | 04110000 | Nemdatine 10mg tablets (Actavis UK Ltd) | Memantine hydrochloride |
| 72855 | 04110000 | Donepezil 5mg tablets (Actavis UK Ltd) | Donepezil hydrochloride |
| 63226 | 04110000 | Prometax 9.5mg/24hours transdermal patches (Sandoz Ltd) | Rivastigmine |
| 37444 | 04110000 | Exelon 4.6mg/24hours transdermal patches (Novartis Pharmaceuticals UK Ltd) | Rivastigmine |
| 60723 | 04110000 | Rivastigmine 6mg capsules (Waymade Healthcare Plc) | Rivastigmine hydrogen tartrate |
| 70069 | 04110000 | Rivastigmine 9.5mg/24hours transdermal patches (A A H Pharmaceuticals Ltd) | Rivastigmine |
| 61476 | 04110000 | Acumor XL 24mg capsules (Viatris UK Healthcare Ltd) | Galantamine hydrobromide |
| 56600 | 04110000 | Donepezil 5mg tablets (Zentiva Pharma UK Ltd) | Donepezil hydrochloride |
| 65501 | 04110000 | Eluden 4.6mg/24hours transdermal patches (Viatris UK Healthcare Ltd) | Rivastigmine |
| 75023 | 04110000 | Almuriva 9.5mg/24hours transdermal patches (Sandoz Ltd) | Rivastigmine |
| 24088 | 04110000 | Reminyl XL 24mg capsules (Takeda UK Ltd) | Galantamine hydrobromide |
| 69859 | 04110000 | Rivastigmine 4.6mg/24hours transdermal patches (Actavis UK Ltd) | Rivastigmine |
| 29288 | 04110000 | Reminyl 4mg/ml oral solution (Takeda UK Ltd) | Galantamine hydrobromide |
| 58780 | 04110000 | Voleze 9.5mg/24hours transdermal patches (Focus Pharmaceuticals Ltd) | Rivastigmine |
| 11752 | 04110000 | Rivastigmine 4.5mg capsules | Rivastigmine hydrogen tartrate |
| 68802 | 04110000 | Donepezil 5mg tablets (Waymade Healthcare Plc) | Donepezil hydrochloride |
| 80977 | 04110000 | Donepezil 5mg tablets (Ranbaxy (UK) Ltd) | Donepezil hydrochloride |
| 68494 | 04110000 | Rivastigmine 6mg capsules (A A H Pharmaceuticals Ltd) | Rivastigmine hydrogen tartrate |
| 11635 | 04110000 | Galantamine 12mg tablets | Galantamine hydrobromide |
| 69564 | 04110000 | Prometax 4.6mg/24hours transdermal patches (Sandoz Ltd) | Rivastigmine |
| 11716 | 04110000 | Exelon 3mg capsules (Novartis Pharmaceuticals UK Ltd) | Rivastigmine hydrogen tartrate |
| 73865 | 04110000 | Donepezil 10mg orodispersible tablets (DE Pharmaceuticals) | Donepezil hydrochloride |
| 66934 | 04110000 | Memantine 10mg orodispersible tablets sugar free | Memantine hydrochloride |
| 53882 | 04110000 | Rivastigmine 2mg/ml oral solution | Rivastigmine hydrogen tartrate |
| 9854 | 04110000 | Reminyl 4mg tablets (Shire Pharmaceuticals Ltd) | Galantamine hydrobromide |
| 70826 | 04110000 | Rivastigmine 9.5mg/24hours transdermal patches (DE Pharmaceuticals) | Rivastigmine |
| 82073 | 04110000 | Memantine 20mg tablets (Niche Generics Ltd) | Memantine hydrochloride |
| 55928 | 04110000 | Exelon 4.5mg capsules (Waymade Healthcare Plc) | Rivastigmine hydrogen tartrate |
| 65761 | 04110000 | Eluden 9.5mg/24hours transdermal patches (Viatris UK Healthcare Ltd) | Rivastigmine |
| 59993 | 04110000 | Galzemic XL 16mg capsules (Zentiva Pharma UK Ltd) | Galantamine hydrobromide |
| 18587 | 04110000 | Reminyl XL 8mg capsules (Takeda UK Ltd) | Galantamine hydrobromide |
| 37188 | 04110000 | Aricept Evess 10mg orodispersible tablets (Eisai Ltd) | Donepezil hydrochloride |
| 86538 | 04110000 | Memantine 20mg tablets (Accord Healthcare Ltd) | Memantine hydrochloride |
| 4597 | 04110000 | Rivastigmine 1.5mg capsules | Rivastigmine hydrogen tartrate |
| 79119 | 04110000 | Donepezil 10mg tablets (Teva UK Ltd) | Donepezil hydrochloride |
| 2931 | 04110000 | Donepezil 10mg tablets | Donepezil hydrochloride |
| 62164 | 04110000 | Alzest 9.5mg/24hours transdermal patches (Dr Reddy's Laboratories (UK) Ltd) | Rivastigmine |
| 66899 | 04110000 | Memantine 20mg orodispersible tablets sugar free | Memantine hydrochloride |
| 63217 | 04110000 | Donepezil 5mg tablets (A A H Pharmaceuticals Ltd) | Donepezil hydrochloride |
| 62925 | 04110000 | Acumor XL 16mg capsules (Viatris UK Healthcare Ltd) | Galantamine hydrobromide |
| 11654 | 04110000 | Galantamine 8mg tablets | Galantamine hydrobromide |
| 56631 | 04110000 | Rivastigmine 13.3mg/24hours transdermal patches | Rivastigmine |
| 37957 | 04110000 | Exelon 9.5mg/24hours transdermal patches (Novartis Pharmaceuticals UK Ltd) | Rivastigmine |
| 75603 | 04110000 | Donepezil 5mg tablets (Macleods Pharma UK Ltd) | Donepezil hydrochloride |
| 63405 | 04110000 | Galsya XL 16mg capsules (Consilient Health Ltd) | Galantamine hydrobromide |
| 84345 | 04110000 | Alzest 13.3mg/24hours transdermal patches (Dr Reddy's Laboratories (UK) Ltd) | Rivastigmine |
| 62780 | 04110000 | Alzest 4.6mg/24hours transdermal patches (Dr Reddy's Laboratories (UK) Ltd) | Rivastigmine |
| 48482 | 04110000 | Galsya XL 8mg capsules (Consilient Health Ltd) | Galantamine hydrobromide |
| 59330 | 04110000 | Voleze 4.6mg/24hours transdermal patches (Advanz Pharma) | Rivastigmine |
| 60192 | 04110000 | Galzemic XL 8mg capsules (Zentiva Pharma UK Ltd) | Galantamine hydrobromide |
| 55720 | 04110000 | Gatalin XL 24mg capsules (Aspire Pharma Ltd) | Galantamine hydrobromide |
| 80637 | 04110000 | Memantine 10mg/ml oral solution sugar free (Teva UK Ltd) | Memantine hydrochloride |
| 71960 | 04110000 | Donepezil 5mg tablets (Accord Healthcare Ltd) | Donepezil hydrochloride |
| 83688 | 04110000 | Erastig 13.3mg/24hours transdermal patches (Teva UK Ltd) | Rivastigmine |
| 63951 | 04110000 | Rivastigmine 9.5mg/24hours transdermal patches (Actavis UK Ltd) | Rivastigmine |
| 83261 | 04110000 | Valios 10mg orodispersible tablets sugar free (Dr Reddy's Laboratories (UK) Ltd) | Memantine hydrochloride |
| 57139 | 04110000 | Ebixa 10mg tablets (DE Pharmaceuticals) | Memantine hydrochloride |
| 61618 | 04110000 | Nemdatine 20mg tablets (Actavis UK Ltd) | Memantine hydrochloride |
| 11837 | 04110000 | Memantine 10mg/ml oral solution sugar free | Memantine hydrochloride |
| 62867 | 04110000 | Gazylan XL 16mg capsules (Teva UK Ltd) | Galantamine hydrobromide |
| 11827 | 04110000 | Rivastigmine 2mg/ml oral solution sugar free | Rivastigmine hydrogen tartrate |
| 79289 | 04110000 | Almuriva 4.6mg/24hours transdermal patches (Sandoz Ltd) | Rivastigmine |
| 65534 | 04110000 | Donepezil 5mg orodispersible tablets sugar free (A A H Pharmaceuticals Ltd) | Donepezil hydrochloride |
| 35088 | 04110000 | Donepezil 10mg orodispersible tablets sugar free | Donepezil hydrochloride |
| 83594 | 04110000 | Reminyl XL 8mg capsules (DE Pharmaceuticals) | Galantamine hydrobromide |
| 70496 | 04110000 | Memantine 10mg soluble tablets sugar free | Memantine hydrochloride |
| 61921 | 04110000 | Luventa XL 24mg capsules (Fontus Health Ltd) | Galantamine hydrobromide |
| 62868 | 04110000 | Gazylan XL 24mg capsules (Teva UK Ltd) | Galantamine hydrobromide |
| 60107 | 04110000 | Donepezil 5mg tablets (Alliance Healthcare (Distribution) Ltd) | Donepezil hydrochloride |
| 72614 | 04110000 | Donepezil 5mg tablets (Viatris UK Healthcare Ltd) | Donepezil hydrochloride |
| 58969 | 04110000 | Rivastigmine 4.6mg/24hours transdermal patches (A A H Pharmaceuticals Ltd) | Rivastigmine |
| 81971 | 04110000 | Valios 20mg orodispersible tablets sugar free (Dr Reddy's Laboratories (UK) Ltd) | Memantine hydrochloride |
| 65573 | 04110000 | Gazylan XL 8mg capsules (Teva UK Ltd) | Galantamine hydrobromide |
| 53842 | 04110000 | Aricept 5mg tablets (Waymade Healthcare Plc) | Donepezil hydrochloride |
| 60493 | 04110000 | Galzemic XL 24mg capsules (Zentiva Pharma UK Ltd) | Galantamine hydrobromide |
| 58937 | 04110000 | Exelon 13.3mg/24hours transdermal patches (Novartis Pharmaceuticals UK Ltd) | Rivastigmine |
| 82072 | 04110000 | Memantine 10mg tablets (Niche Generics Ltd) | Memantine hydrochloride |
| 83674 | 04110000 | Memantine 10mg tablets (Viatris UK Healthcare Ltd) | Memantine hydrochloride |
| 70503 | 04110000 | Memantine 20mg soluble tablets sugar free | Memantine hydrochloride |
| 69971 | 04110000 | Memantine 10mg/ml oral solution sugar free (Zentiva Pharma UK Ltd) | Memantine hydrochloride |
| 39363 | 04110000 | Ebixa 20mg tablets (Lundbeck Ltd) | Memantine hydrochloride |
| 74058 | 04110000 | Aricept 10mg tablets (Waymade Healthcare Plc) | Donepezil hydrochloride |
| 36848 | 04110000 | Aricept Evess 5mg orodispersible tablets (Eisai Ltd) | Donepezil hydrochloride |
| 65333 | 04110000 | Memantine 10mg/ml oral solution sugar free (Alliance Healthcare (Distribution) Ltd) | Memantine hydrochloride |
| 20140 | 04110000 | Reminyl XL 16mg capsules (Takeda UK Ltd) | Galantamine hydrobromide |
| 56709 | 04110000 | Gatalin XL 16mg capsules (Aspire Pharma Ltd) | Galantamine hydrobromide |
| 67593 | 04110000 | Donepezil 10mg tablets (Zentiva Pharma UK Ltd) | Donepezil hydrochloride |
| 59871 | 04110000 | Donepezil 10mg/5ml oral suspension | Donepezil hydrochloride |
| 2930 | 04110000 | Donepezil 5mg tablets | Donepezil hydrochloride |
| 69595 | 04110000 | Marixino 20mg tablets (Consilient Health Ltd) | Memantine hydrochloride |
| 7361 | 04110000 | Galantamine 24mg modified-release capsules | Galantamine hydrobromide |
| 56421 | 04110000 | Gatalin XL 8mg capsules (Aspire Pharma Ltd) | Galantamine hydrobromide |
| 18800 | 04110000 | Ebixa 10mg tablets (Lundbeck Ltd) | Memantine hydrochloride |
| 61676 | 04110000 | Donepezil 1mg/ml oral solution sugar free | Donepezil hydrochloride |
| 82310 | 04110000 | Donepezil 10mg tablets (Pfizer Ltd) | Donepezil hydrochloride |
| 83370 | 04110000 | Memantine 10mg tablets (Dawa Ltd) | Memantine hydrochloride |
| 71187 | 04110000 | Memantine 10mg tablets (A A H Pharmaceuticals Ltd) | Memantine hydrochloride |
| 5247 | 04110000 | Aricept 10mg tablets (Eisai Ltd) | Donepezil hydrochloride |
| 5400 | 04110000 | Aricept 5mg tablets (Eisai Ltd) | Donepezil hydrochloride |
| 72953 | 04110000 | Donepezil 10mg tablets (Mawdsley-Brooks & Company Ltd) | Donepezil hydrochloride |
| 58947 | 04110000 | Donepezil 10mg tablets (Accord Healthcare Ltd) | Donepezil hydrochloride |
| 57171 | 04110000 | Erastig 9.5mg/24hours transdermal patches (Teva UK Ltd) | Rivastigmine |
| 63360 | 04110000 | Luventa XL 16mg capsules (Fontus Health Ltd) | Galantamine hydrobromide |
| 14309 | 04110000 | Galantamine 16mg modified-release capsules | Galantamine hydrobromide |
| 39362 | 04110000 | Ebixa tablets treatment initiation pack (Lundbeck Ltd) | Memantine Hydrochloride |
| 10187 | 04110000 | Galantamine 4mg tablets | Galantamine hydrobromide |
| 79120 | 04110000 | Donepezil 10mg tablets (Phoenix Healthcare Distribution Ltd) | Donepezil hydrochloride |
| 48015 | 04110000 | Galsya XL 24mg capsules (Consilient Health Ltd) | Galantamine hydrobromide |
| 35179 | 04110000 | Donepezil 5mg orodispersible tablets sugar free | Donepezil hydrochloride |
| 18062 | 04110000 | Reminyl 8mg tablets (Shire Pharmaceuticals Ltd) | Galantamine hydrobromide |
| 57627 | 04110000 | Erastig 4.6mg/24hours transdermal patches (Teva UK Ltd) | Rivastigmine |
| 58709 | 04110000 | Donepezil 10mg tablets (A A H Pharmaceuticals Ltd) | Donepezil hydrochloride |
| 85105 | 04110000 | Memantine 10mg/ml oral solution sugar free (Rosemont Pharmaceuticals Ltd) | Memantine hydrochloride |
| 9786 | 04110000 | Rivastigmine 6mg capsules | Rivastigmine hydrogen tartrate |
| 20404 | 04110000 | Exelon 4.5mg capsules (Novartis Pharmaceuticals UK Ltd) | Rivastigmine hydrogen tartrate |
| 6225 | 04110000 | Memantine 10mg tablets | Memantine hydrochloride |
| 82302 | 04110000 | Donepezil 5mg tablets (Pfizer Ltd) | Donepezil hydrochloride |
| 79432 | 04110000 | Galantamine 8mg tablets (A A H Pharmaceuticals Ltd) | Galantamine hydrobromide |
| 64982 | 04110000 | Memantine 20mg tablets (Teva UK Ltd) | Memantine hydrochloride |
| 37132 | 04110000 | Rivastigmine 9.5mg/24hours transdermal patches | Rivastigmine |
| 18556 | 04110000 | Exelon 2mg/ml oral solution (Novartis Pharmaceuticals UK Ltd) | Rivastigmine hydrogen tartrate |
| 79754 | 04110000 | Donepezil 10mg tablets (Actavis UK Ltd) | Donepezil hydrochloride |
| 48442 | 04110000 | Donepezil 5mg orodispersible tablets | Donepezil hydrochloride |

Source: CPRD GOLD Code Browser Version 3.0.0 (2)

## Dementia type codes

| **Read code** | **Med code** | **Description** | **Type** |
| --- | --- | --- | --- |
| F110.00 | 1917 | Alzheimer's disease | Alzheimer’s |
| Eu00.00 | 7664 | [X]Dementia in Alzheimer's disease | Alzheimer’s |
| Eu00z11 | 8195 | [X]Alzheimer's dementia unspec | Alzheimer’s |
| Eu00112 | 11379 | [X]Senile dementia, Alzheimer’s type | Alzheimer’s |
| F110000 | 16797 | Alzheimer's disease with early onset | Alzheimer’s |
| Eu00z00 | 29386 | [X]Dementia in Alzheimer's disease, unspecified | Alzheimer’s |
| Eu00200 | 30706 | [X]Dementia in Alzheimer's dis, atypical or mixed type | Alzheimer’s |
| F110100 | 32057 | Alzheimer's disease with late onset | Alzheimer’s |
| Eu00100 | 38678 | [X]Dementia in Alzheimer's disease with late onset | Alzheimer’s |
| Eu00113 | 43346 | [X]Primary degen dementia of Alzheimer's type, senile onset | Alzheimer’s |
| Eu00111 | 46762 | [X]Alzheimer's disease type 1 | Alzheimer’s |
| Eu01111 | 55838 | [X]Predominantly cortical dementia | Alzheimer’s |
| Fyu3000 | 59122 | [X]Other Alzheimer's disease | Alzheimer’s |
| Eu00013 | 61528 | [X]Alzheimer's disease type 2 | Alzheimer’s |
| Eu01.00 | 6578 | [X]Vascular dementia | VaD |
| E004.11 | 8634 | Multi infarct dementia | VaD |
| Eu01200 | 8934 | [X]Subcortical vascular dementia | VaD |
| Eu01.11 | 9565 | [X]Arteriosclerotic dementia | VaD |
| Eu01100 | 11175 | [X]Multi-infarct dementia | VaD |
| Eu01z00 | 19393 | [X]Vascular dementia, unspecified | VaD |
| E004.00 | 19477 | Arteriosclerotic dementia | VaD |
| E004z00 | 42279 | Arteriosclerotic dementia NOS | VaD |
| E004000 | 43089 | Uncomplicated arteriosclerotic dementia | VaD |
| E004300 | 43292 | Arteriosclerotic dementia with depression | VaD |
| Eu01000 | 46488 | [X]Vascular dementia of acute onset | VaD |
| Eu01y00 | 55313 | [X]Other vascular dementia | VaD |
| E004200 | 55467 | Arteriosclerotic dementia with paranoia | VaD |
| E004100 | 56912 | Arteriosclerotic dementia with delirium | VaD |
| F116.00 | 7572 | Lewy body disease | DLB |
| Eu02500 | 26270 | [X]Lewy body dementia | DLB |
| Eu01300 | 31016 | [X]Mixed cortical and subcortical vascular dementia | Mixed |
| Eu10711 | 26323 | [X]Alcoholic dementia NOS | Alcoholic |
| E012.11 | 27342 | Alcoholic dementia NOS | Alcoholic |
| E00..11 | 1916 | Senile dementia | Unspecified |
| Eu02z14 | 4357 | [X] Senile dementia NOS | Unspecified |
| Eu02z00 | 4693 | [X] Unspecified dementia | Unspecified |
| E000.00 | 7323 | Uncomplicated senile dementia | Unspecified |
| E002000 | 18386 | Senile dementia with paranoia | Unspecified |
| E002100 | 21887 | Senile dementia with depression | Unspecified |
| Eu02z16 | 27759 | [X] Senile dementia, depressed or paranoid type | Unspecified |
| Eu02z13 | 34944 | [X] Primary degenerative dementia NOS | Unspecified |
| E003.00 | 37015 | Senile dementia with delirium | Unspecified |
| E002z00 | 41089 | Senile dementia with depressive or paranoid features NOS | Unspecified |
| E002.00 | 44674 | Senile dementia with depressive or paranoid features | Unspecified |
| Eu02y00 | 64267 | [X]Dementia in other specified diseases classif elsewhere | Unspecified |

Source: Kontopantelis et al (1)

# Table S2: Code lists for other long term conditions

## Type 1 diabetes codes

| **Read code** | **Med code** | **Description** |
| --- | --- | --- |
| C100011 | 1038 | Insulin dependent diabetes mellitus |
| C10E.00 | 1549 | Type 1 diabetes mellitus |
| C108.00 | 1647 | Insulin dependent diabetes mellitus |
| 66AJ100 | 2478 | Brittle diabetes |
| C108700 | 6509 | Insulin dependent diabetes mellitus with retinopathy |
| C108800 | 6791 | Insulin dependent diabetes mellitus - poor control |
| C10ED00 | 10418 | Type 1 diabetes mellitus with nephropathy |
| C10EM00 | 10692 | Type 1 diabetes mellitus with ketoacidosis |
| C10E.11 | 12455 | Type I diabetes mellitus |
| C108F11 | 17545 | Type I diabetes mellitus with diabetic cataract |
| C108.12 | 17858 | Type 1 diabetes mellitus |
| C108J12 | 18230 | Type 1 diabetes mellitus with neuropathic arthropathy |
| C10E700 | 18387 | Type 1 diabetes mellitus with retinopathy |
| C108.11 | 18505 | IDDM-Insulin dependent diabetes mellitus |
| C10EH00 | 18642 | Type 1 diabetes mellitus with arthropathy |
| C10E500 | 18683 | Type 1 diabetes mellitus with ulcer |
| C10EP00 | 22871 | Type 1 diabetes mellitus with exudative maculopathy |
| C108.13 | 24423 | Type I diabetes mellitus |
| C108B00 | 24694 | Insulin dependent diabetes mellitus with mononeuropathy |
| C108400 | 26855 | Unstable insulin dependent diabetes mellitus |
| C10EL00 | 30294 | Type 1 diabetes mellitus with persistent microalbuminuria |
| C10E800 | 35288 | Type 1 diabetes mellitus - poor control |
| C108711 | 38161 | Type I diabetes mellitus with retinopathy |
| C10EE00 | 39070 | Type 1 diabetes mellitus with hypoglycaemic coma |
| C10EN00 | 40837 | Type 1 diabetes mellitus with ketoacidotic coma |
| C108712 | 41049 | Type 1 diabetes mellitus with retinopathy |
| C108E11 | 42729 | Type I diabetes mellitus with hypoglycaemic coma |
| C10E200 | 42831 | Type 1 diabetes mellitus with neurological complications |
| C10E400 | 43921 | Unstable type 1 diabetes mellitus |
| C108F00 | 44260 | Insulin dependent diabetes mellitus with diabetic cataract |
| C108E00 | 44440 | Insulin dependent diabetes mellitus with hypoglycaemic coma |
| C108500 | 44443 | Insulin dependent diabetes mellitus with ulcer |
| C10E312 | 45276 | Insulin dependent diabetes mellitus with multiple complicat |
| C108812 | 45914 | Type 1 diabetes mellitus - poor control |
| C10EC00 | 46301 | Type 1 diabetes mellitus with polyneuropathy |
| C108811 | 46850 | Type I diabetes mellitus - poor control |
| C108000 | 46963 | Insulin-dependent diabetes mellitus with renal complications |
| C10E000 | 47582 | Type 1 diabetes mellitus with renal complications |
| C10E100 | 47649 | Type 1 diabetes mellitus with ophthalmic complications |
| C10E300 | 47650 | Type 1 diabetes mellitus with multiple complications |
| C108211 | 49146 | Type I diabetes mellitus with neurological complications |
| C108100 | 49276 | Insulin-dependent diabetes mellitus with ophthalmic comps |
| C10EF00 | 49554 | Type 1 diabetes mellitus with diabetic cataract |
| C10E411 | 49949 | Unstable type I diabetes mellitus |
| L180500 | 50960 | Pre-existing diabetes mellitus, insulin-dependent |
| C10E.12 | 51261 | Insulin dependent diabetes mellitus |
| C108511 | 51957 | Type I diabetes mellitus with ulcer |
| C108300 | 52104 | Insulin dependent diabetes mellitus with multiple complicatn |
| C108200 | 52283 | Insulin-dependent diabetes mellitus with neurological comps |
| C10EJ00 | 54008 | Type 1 diabetes mellitus with neuropathic arthropathy |
| C10E412 | 54600 | Unstable insulin dependent diabetes mellitus |
| C10EQ00 | 55239 | Type 1 diabetes mellitus with gastroparesis |
| C108A00 | 56448 | Insulin-dependent diabetes without complication |
| C108D00 | 57621 | Insulin dependent diabetes mellitus with nephropathy |
| C108411 | 60107 | Unstable type I diabetes mellitus |
| C108J11 | 60208 | Type I diabetes mellitus with neuropathic arthropathy |
| C108600 | 60499 | Insulin dependent diabetes mellitus with gangrene |
| C108011 | 61344 | Type I diabetes mellitus with renal complications |
| C108212 | 61829 | Type 1 diabetes mellitus with neurological complications |
| C10EM11 | 62209 | Type I diabetes mellitus with ketoacidosis |
| C108H11 | 62352 | Type I diabetes mellitus with arthropathy |
| C10EA11 | 62613 | Type I diabetes mellitus without complication |
| C108H00 | 65616 | Insulin dependent diabetes mellitus with arthropathy |
| C10EN11 | 66145 | Type I diabetes mellitus with ketoacidotic coma |
| C108D11 | 66872 | Type I diabetes mellitus with nephropathy |
| C10EB00 | 68105 | Type 1 diabetes mellitus with mononeuropathy |
| C108512 | 68390 | Type 1 diabetes mellitus with ulcer |
| C107300 | 69124 | IDDM with peripheral circulatory disorder |
| C10EA00 | 69676 | Type 1 diabetes mellitus without complication |
| C10E600 | 69993 | Type 1 diabetes mellitus with gangrene |
| C108E12 | 70766 | Type 1 diabetes mellitus with hypoglycaemic coma |
| C10E812 | 72702 | Insulin dependent diabetes mellitus - poor control |
| C10E311 | 91942 | Type I diabetes mellitus with multiple complications |
| C10EC11 | 91943 | Type I diabetes mellitus with polyneuropathy |
| C10EG00 | 93468 | Type 1 diabetes mellitus with peripheral angiopathy |
| C10E712 | 93875 | Insulin dependent diabetes mellitus with retinopathy |
| C10E511 | 93878 | Type I diabetes mellitus with ulcer |
| C10E711 | 95343 | Type I diabetes mellitus with retinopathy |
| C108A11 | 95992 | Type I diabetes mellitus without complication |
| C108012 | 21983 | Type 1 diabetes mellitus with renal complications |
| C108C00 | 41716 | Insulin dependent diabetes mellitus with polyneuropathy |

Source: Kuan et al (3)

## Type 2 diabetes codes

| **Read code** | **Med code** | **Description** |
| --- | --- | --- |
| C100112 | 506 | Non-insulin dependent diabetes mellitus |
| C10FJ00 | 1407 | Insulin treated Type 2 diabetes mellitus |
| C109.00 | 4513 | Non-insulin dependent diabetes mellitus |
| C109.11 | 5884 | NIDDM - Non-insulin dependent diabetes mellitus |
| C109700 | 8403 | Non-insulin dependent diabetes mellitus - poor control |
| C10FC00 | 12640 | Type 2 diabetes mellitus with nephropathy |
| C10F500 | 12736 | Type 2 diabetes mellitus with gangrene |
| C109600 | 17262 | Non-insulin-dependent diabetes mellitus with retinopathy |
| C109.12 | 17859 | Type 2 diabetes mellitus |
| C109G11 | 18143 | Type II diabetes mellitus with arthropathy |
| C109012 | 18209 | Type 2 diabetes mellitus with renal complications |
| C109.13 | 18219 | Type II diabetes mellitus |
| C10FM00 | 18390 | Type 2 diabetes mellitus with persistent microalbuminuria |
| C10FB00 | 18425 | Type 2 diabetes mellitus with polyneuropathy |
| C10F600 | 18496 | Type 2 diabetes mellitus with retinopathy |
| C10F000 | 18777 | Type 2 diabetes mellitus with renal complications |
| C109H00 | 40962 | Non-insulin dependent d m with neuropathic arthropathy |
| C10F.11 | 22884 | Type II diabetes mellitus |
| C109711 | 24458 | Type II diabetes mellitus - poor control |
| C109G00 | 24693 | Non-insulin dependent diabetes mellitus with arthropathy |
| C109C12 | 24836 | Type 2 diabetes mellitus with nephropathy |
| C10FQ00 | 25591 | Type 2 diabetes mellitus with exudative maculopathy |
| C10F700 | 25627 | Type 2 diabetes mellitus - poor control |
| C10FL00 | 26054 | Type 2 diabetes mellitus with persistent proteinuria |
| C109900 | 29979 | Non-insulin-dependent diabetes mellitus without complication |
| C10FN00 | 32627 | Type 2 diabetes mellitus with ketoacidosis |
| C10F200 | 34268 | Type 2 diabetes mellitus with neurological complications |
| C109400 | 34912 | Non-insulin dependent diabetes mellitus with ulcer |
| C10FH00 | 35385 | Type 2 diabetes mellitus with neuropathic arthropathy |
| C109J11 | 37648 | Insulin treated non-insulin dependent diabetes mellitus |
| C10FF00 | 37806 | Type 2 diabetes mellitus with peripheral angiopathy |
| C109500 | 40401 | Non-insulin dependent diabetes mellitus with gangrene |
| C10F.00 | 758 | Type 2 diabetes mellitus |
| C109612 | 42762 | Type 2 diabetes mellitus with retinopathy |
| C10F311 | 43227 | Type II diabetes mellitus with multiple complications |
| C109D00 | 43785 | Non-insulin dependent diabetes mellitus with hypoglyca coma |
| C109E12 | 44779 | Type 2 diabetes mellitus with diabetic cataract |
| C10FE00 | 44982 | Type 2 diabetes mellitus with diabetic cataract |
| C109B00 | 45467 | Non-insulin dependent diabetes mellitus with polyneuropathy |
| C109712 | 45913 | Type 2 diabetes mellitus - poor control |
| C109212 | 45919 | Type 2 diabetes mellitus with neurological complications |
| C109512 | 46150 | Type 2 diabetes mellitus with gangrene |
| C10FD00 | 46917 | Type 2 diabetes mellitus with hypoglycaemic coma |
| C10F711 | 47315 | Type II diabetes mellitus - poor control |
| C10F100 | 47321 | Type 2 diabetes mellitus with ophthalmic complications |
| C109B11 | 47409 | Type II diabetes mellitus with polyneuropathy |
| C109H11 | 47816 | Type II diabetes mellitus with neuropathic arthropathy |
| C10F900 | 47954 | Type 2 diabetes mellitus without complication |
| C109E11 | 48192 | Type II diabetes mellitus with diabetic cataract |
| C10F400 | 49074 | Type 2 diabetes mellitus with ulcer |
| C10F611 | 49655 | Type II diabetes mellitus with retinopathy |
| C109G12 | 49869 | Type 2 diabetes mellitus with arthropathy |
| C109011 | 50225 | Type II diabetes mellitus with renal complications |
| C109100 | 50429 | Non-insulin-dependent diabetes mellitus with ophthalm comps |
| C10FB11 | 50527 | Type II diabetes mellitus with polyneuropathy |
| C109A11 | 50813 | Type II diabetes mellitus with mononeuropathy |
| C10FP00 | 51756 | Type 2 diabetes mellitus with ketoacidotic coma |
| C109000 | 52303 | Non-insulin-dependent diabetes mellitus with renal comps |
| C10F911 | 53392 | Type II diabetes mellitus without complication |
| C109F11 | 54899 | Type II diabetes mellitus with peripheral angiopathy |
| C109411 | 55075 | Type II diabetes mellitus with ulcer |
| C109200 | 55842 | Non-insulin-dependent diabetes mellitus with neuro comps |
| C109D11 | 56268 | Type II diabetes mellitus with hypoglycaemic coma |
| C107400 | 56803 | NIDDM with peripheral circulatory disorder |
| C10F011 | 57278 | Type II diabetes mellitus with renal complications |
| C109611 | 58604 | Type II diabetes mellitus with retinopathy |
| C10FG00 | 59253 | Type 2 diabetes mellitus with arthropathy |
| C109C00 | 59365 | Non-insulin dependent diabetes mellitus with nephropathy |
| C109111 | 59725 | Type II diabetes mellitus with ophthalmic complications |
| C109F12 | 60699 | Type 2 diabetes mellitus with peripheral angiopathy |
| C10FL11 | 60796 | Type II diabetes mellitus with persistent proteinuria |
| C109D12 | 61071 | Type 2 diabetes mellitus with hypoglycaemic coma |
| C109511 | 62107 | Type II diabetes mellitus with gangrene |
| C109300 | 62146 | Non-insulin-dependent diabetes mellitus with multiple comps |
| C10FA00 | 62674 | Type 2 diabetes mellitus with mononeuropathy |
| C10FR00 | 63690 | Type 2 diabetes mellitus with gastroparesis |
| C109C11 | 64571 | Type II diabetes mellitus with nephropathy |
| C10FJ11 | 64668 | Insulin treated Type II diabetes mellitus |
| C10F300 | 65267 | Type 2 diabetes mellitus with multiple complications |
| C109412 | 65704 | Type 2 diabetes mellitus with ulcer |
| C109H12 | 66965 | Type 2 diabetes mellitus with neuropathic arthropathy |
| C109211 | 67905 | Type II diabetes mellitus with neurological complications |
| C109E00 | 69278 | Non-insulin depend diabetes mellitus with diabetic cataract |
| C109112 | 70316 | Type 2 diabetes mellitus with ophthalmic complications |
| C109A00 | 72320 | Non-insulin dependent diabetes mellitus with mononeuropathy |
| C10FM11 | 85991 | Type II diabetes mellitus with persistent microalbuminuria |
| C10F411 | 91646 | Type II diabetes mellitus with ulcer |
| C10FE11 | 93727 | Type II diabetes mellitus with diabetic cataract |
| C10FA11 | 95351 | Type II diabetes mellitus with mononeuropathy |

Source: Kuan et al (3)

## Cardiovascular disease codes

| **Read code** | **Med code** | **Description** |
| --- | --- | --- |
| G3...00 | 240 | Ischaemic heart disease |
| G30..00 | 241 | Acute myocardial infarction |
| G300.00 | 12139 | Acute anterolateral infarction |
| G301.00 | 5387 | Other specified anterior myocardial infarction |
| G301000 | 40429 | Acute anteroapical infarction |
| G301100 | 17872 | Acute anteroseptal infarction |
| G30..12 | 2491 | Coronary thrombosis |
| G30..13 | 30421 | Cardiac rupture following myocardial infarction (MI) |
| G30..15 | 1677 | MI - acute myocardial infarction |
| G30..16 | 13571 | Thrombosis - coronary |
| G30..17 | 17689 | Silent myocardial infarction |
| G301z00 | 14897 | Anterior myocardial infarction NOS |
| G302.00 | 8935 | Acute inferolateral infarction |
| G303.00 | 29643 | Acute inferoposterior infarction |
| G304.00 | 23892 | Posterior myocardial infarction NOS |
| G305.00 | 14898 | Lateral myocardial infarction NOS |
| G306.00 | 63467 | True posterior myocardial infarction |
| G307.00 | 3704 | Acute subendocardial infarction |
| G307000 | 9507 | Acute non-Q wave infarction |
| G307100 | 10562 | Acute non-ST segment elevation myocardial infarction |
| G308.00 | 1678 | Inferior myocardial infarction NOS |
| G309.00 | 30330 | Acute Q-wave infarct |
| G30B.00 | 32854 | Acute posterolateral myocardial infarction |
| G30X.00 | 29758 | Acute transmural myocardial infarction of unspecif site |
| G30X000 | 12229 | Acute ST segment elevation myocardial infarction |
| G30y.00 | 34803 | Other acute myocardial infarction |
| G30y100 | 62626 | Acute papillary muscle infarction |
| G30y200 | 41221 | Acute septal infarction |
| G30yz00 | 46017 | Other acute myocardial infarction NOS |
| G30z.00 | 14658 | Acute myocardial infarction NOS |
| G31..00 | 27951 | Other acute and subacute ischaemic heart disease |
| G310.00 | 23579 | Postmyocardial infarction syndrome |
| G310.11 | 15661 | Dressler's syndrome |
| G3...11 | 24783 | Arteriosclerotic heart disease |
| G311.00 | 36523 | Preinfarction syndrome |
| G311100 | 7347 | Unstable angina |
| G311.11 | 4656 | Crescendo angina |
| G311.12 | 39655 | Impending infarction |
| G311.13 | 1431 | Unstable angina |
| G311.14 | 19655 | Angina at rest |
| G311200 | 17307 | Angina at rest |
| G311300 | 34328 | Refractory angina |
| G311500 | 11983 | Acute coronary syndrome |
| G311z00 | 54251 | Preinfarction syndrome NOS |
| G3...12 | 20416 | Atherosclerotic heart disease |
| G3...13 | 1792 | IHD - Ischaemic heart disease |
| G31y.00 | 9413 | Other acute and subacute ischaemic heart disease |
| G31y000 | 9276 | Acute coronary insufficiency |
| G31y200 | 39693 | Subendocardial ischaemia |
| G31y300 | 21844 | Transient myocardial ischaemia |
| G31yz00 | 27977 | Other acute and subacute ischaemic heart disease NOS |
| G33..00 | 1430 | Angina pectoris |
| G330.00 | 20095 | Angina decubitus |
| G330000 | 18125 | Nocturnal angina |
| G330z00 | 29902 | Angina decubitus NOS |
| G331.00 | 12986 | Prinzmetal's angina |
| G331.11 | 11048 | Variant angina pectoris |
| G332.00 | 36854 | Coronary artery spasm |
| G33z.00 | 25842 | Angina pectoris NOS |
| G33z000 | 66388 | Status anginosus |
| G33z100 | 54535 | Stenocardia |
| G33z200 | 7696 | Syncope anginosa |
| G33z300 | 1414 | Angina on effort |
| G33z400 | 32450 | Ischaemic chest pain |
| G33z500 | 9555 | Post infarct angina |
| G33z600 | 26863 | New onset angina |
| G33z700 | 12804 | Stable angina |
| G33zz00 | 28554 | Angina pectoris NOS |
| G34..00 | 28138 | Other chronic ischaemic heart disease |
| G340.00 | 5413 | Coronary atherosclerosis |
| G340000 | 3999 | Single coronary vessel disease |
| G340100 | 5254 | Double coronary vessel disease |
| G340.11 | 1655 | Triple vessel disease of the heart |
| G340.12 | 1344 | Coronary artery disease |
| G342.00 | 36609 | Atherosclerotic cardiovascular disease |
| G343.00 | 7320 | Ischaemic cardiomyopathy |
| G344.00 | 29421 | Silent myocardial ischaemia |
| G34y.00 | 34633 | Other specified chronic ischaemic heart disease |
| G34y000 | 24540 | Chronic coronary insufficiency |
| G34y100 | 23078 | Chronic myocardial ischaemia |
| G34yz00 | 35713 | Other specified chronic ischaemic heart disease NOS |
| G34z.00 | 15754 | Other chronic ischaemic heart disease NOS |
| G34z000 | 18889 | Asymptomatic coronary heart disease |
| G35..00 | 18842 | Subsequent myocardial infarction |
| G350.00 | 45809 | Subsequent myocardial infarction of anterior wall |
| G351.00 | 38609 | Subsequent myocardial infarction of inferior wall |
| G353.00 | 72562 | Subsequent myocardial infarction of other sites |
| G35X.00 | 46166 | Subsequent myocardial infarction of unspecified site |
| G3y..00 | 22383 | Other specified ischaemic heart disease |
| G3z..00 | 1676 | Ischaemic heart disease NOS |
| G70..00 | 5640 | Atherosclerosis |
| G700.00 | 1318 | Aortic atherosclerosis |
| G73..00 | 5943 | Other peripheral vascular disease |
| G73..12 | 1826 | Ischaemia of legs |
| G73y.00 | 38907 | Other specified peripheral vascular disease |
| G73yz00 | 4325 | Other specified peripheral vascular disease NOS |
| G73z.00 | 3530 | Peripheral vascular disease NOS |
| G73zz00 | 2760 | Peripheral vascular disease NOS |
| G742z00 | 15302 | Peripheral arterial embolism and thrombosis NOS |
| Gyu3.00 | 52517 | [X]Ischaemic heart diseases |
| Gyu3300 | 47637 | [X]Other forms of chronic ischaemic heart disease |
| Gyu3600 | 99991 | [X]Subsequent myocardial infarction of unspecified site |
| Gyu7400 | 73961 | [X]Other specified peripheral vascular diseases |

Source: Wright et al (4)

## History of stroke codes

| **Read code** | **Med code** | **Description** |
| --- | --- | --- |
| 14A7.11 | 6305 | H/O: CVA |
| 14A7.12 | 5871 | H/O: stroke |
| 661M700 | 107195 | Stroke self-management plan agreed |
| 14A7.00 | 34135 | H/O: CVA/stroke |
| 661N700 | 109743 | Stroke self-management plan review |
| ZV12511 | 19348 | [V]Personal history of stroke |
| 662M100 | 105100 | Stroke 6 month review |
| 662M.00 | 10792 | Stroke monitoring |
| ZV12512 | 7138 | [V]Personal history of cerebrovascular accident (CVA) |
| 14AK.00 | 66873 | H/O: Stroke in last year |
| 662e.00 | 18686 | Stroke/CVA annual review |
| 662e.11 | 107886 | Stroke annual review |

Source: Kuan et al (3)

## Cerebrovascular disease codes

| **Read code** | **Med code** | **Description** |
| --- | --- | --- |
| G641.11 | 34758 | Cerebral embolus |
| G6...00 | 2418 | Cerebrovascular disease |
| G656.00 | 10794 | Vertebrobasilar insufficiency |
| G67..00 | 13577 | Other cerebrovascular disease |
| Gyu6600 | 92036 | [X]Occlusion and stenosis of other cerebral arteries |
| G63..12 | 63830 | Stenosis of precerebral arteries |
| G65zz00 | 15788 | Transient cerebral ischaemia NOS |
| G68..00 | 23361 | Late effects of cerebrovascular disease |
| G653.00 | 44765 | Carotid artery syndrome hemispheric |
| Gyu6F00 | 96630 | [X]Intracerebral haemorrhage in hemisphere, unspecified |
| G677200 | 65770 | Occlusion and stenosis of posterior cerebral artery |
| G70y011 | 22677 | Carotid artery disease |
| Gyu6700 | 225202 | [X]Other specified cerebrovascular diseases |
| G677400 | 71274 | Occlusion+stenosis of multiple and bilat cerebral arteries |
| G679.00 | 98188 | Small vessel cerebrovascular disease |
| G634.00 | 2652 | Carotid artery stenosis |
| G651.00 | 33377 | Vertebral artery syndrome |
| G631.11 | 2156 | Stenosis, carotid artery |
| G63z.00 | 71585 | Precerebral artery occlusion NOS |
| G60..00 | 1786 | Subarachnoid haemorrhage |
| L440000 | 67640 | Puerperal cerebrovascular disorder unspecified |
| G65z100 | 16507 | Intermittent cerebral ischaemia |
| G65..13 | 2417 | Vertebro-basilar insufficiency |
| G677100 | 57527 | Occlusion and stenosis of anterior cerebral artery |
| G64..00 | 8837 | Cerebral arterial occlusion |
| G633.00 | 98642 | Multiple and bilateral precerebral arterial occlusion |
| G677300 | 55602 | Occlusion and stenosis of cerebellar arteries |
| G6z..00 | 10062 | Cerebrovascular disease NOS |
| Gyu6.00 | 73901 | [X]Cerebrovascular diseases |
| G67y.00 | 34117 | Other cerebrovascular disease OS |
| G671000 | 70536 | Acute cerebrovascular insufficiency NOS |
| G671z00 | 12555 | Generalised ischaemic cerebrovascular disease NOS |
| Gyu6500 | 90572 | [X]Occlusion and stenosis of other precerebral arteries |
| G67z.00 | 37493 | Other cerebrovascular disease NOS |
| G6y..00 | 51311 | Other specified cerebrovascular disease |

Source: Metcalfe et al (5)

## Chronic obstructive pulmonary disease codes

| **Read code** | **Med code** | **Description** |
| --- | --- | --- |
| H3z..11 | 10980 | Chronic obstructive pulmonary disease NOS |
| H36..00 | 1446 | Mild chronic obstructive pulmonary disease |
| H321.00 | 106650 | Panlobular emphysema |
| H31yz00 | 21061 | Other chronic bronchitis NOS |
| H32yz00 | 99536 | Other emphysema NOS |
| H320100 | 10863 | Zonal bullous emphysema |
| H31y.00 | 40788 | Other chronic bronchitis |
| H32y000 | 63479 | Acute vesicular emphysema |
| H312100 | 12166 | Emphysematous bronchitis |
| H3...11 | 794 | Chronic obstructive airways disease |
| H322.00 | 40159 | Centrilobular emphysema |
| H310000 | 9876 | Chronic catarrhal bronchitis |
| H3z..00 | 46578 | Chronic obstructive airways disease NOS |
| H320200 | 15157 | Giant bullous emphysema |
| H312011 | 63216 | Chronic wheezy bronchitis |
| Hyu3000 | 1001 | [X]Other emphysema |
| H38..00 | 66058 | Severe chronic obstructive pulmonary disease |
| H32y100 | 26306 | Atrophic (senile) emphysema |
| H3...00 | 24248 | Chronic obstructive pulmonary disease |
| H32z.00 | 44525 | Emphysema NOS |
| Hyu3100 | 64721 | [X]Other specified chronic obstructive pulmonary disease |
| H3A..00 | 93568 | End stage chronic obstructive airways disease |
| H39..00 | 68662 | Very severe chronic obstructive pulmonary disease |
| H320000 | 5710 | Segmental bullous emphysema |
| H32y111 | 33450 | Acute interstitial emphysema |
| H583200 | 70787 | Eosinophilic bronchitis |
| H311100 | 37247 | Fetid chronic bronchitis |
| H310.00 | 59263 | Simple chronic bronchitis |
| H31z.00 | 27819 | Chronic bronchitis NOS |
| H312200 | 14798 | Acute exacerbation of chronic obstructive airways disease |
| H31..00 | 15626 | Chronic bronchitis |
| H320300 | 25603 | Bullous emphysema with collapse |
| H312000 | 7884 | Chronic asthmatic bronchitis |
| H32y.00 | 5798 | Other emphysema |
| H31y100 | 67040 | Chronic tracheobronchitis |
| H3y..11 | 3243 | Other specified chronic obstructive pulmonary disease |
| H310z00 | 11150 | Simple chronic bronchitis NOS |
| H320z00 | 65733 | Chronic bullous emphysema NOS |
| H311.00 | 23492 | Mucopurulent chronic bronchitis |
| H311000 | 26125 | Purulent chronic bronchitis |
| H3y1.00 | 92955 | Chron obstruct pulmonary dis wth acute exacerbation, unspec |
| H32y200 | 68066 | MacLeod's unilateral emphysema |
| H3y0.00 | 45089 | Chronic obstruct pulmonary dis with acute lower resp infectn |
| H312z00 | 16410 | Obstructive chronic bronchitis NOS |
| H320.00 | 10802 | Chronic bullous emphysema |
| H312.00 | 56860 | Obstructive chronic bronchitis |
| H37..00 | 60188 | Moderate chronic obstructive pulmonary disease |
| H3y..00 | 104608 | Other specified chronic obstructive airways disease |
| H311z00 | 998 | Mucopurulent chronic bronchitis NOS |
| H32..00 | 61118 | Emphysema |
| H313.00 | 37959 | Mixed simple and mucopurulent chronic bronchitis |

Source: Kuan et al (3)

## Chronic kidney disease codes

| **Read code** | **Med code** | **Description** |
| --- | --- | --- |
| 1Z15.00 | 94965 | Chronic kidney disease stage 3A |
| K05..12 | 53852 | End stage renal failure |
| K05..00 | 512 | Chronic renal failure |
| 1Z14.00 | 12585 | Chronic kidney disease stage 5 |
| K100500 | 48855 | Chronic obstructive pyelonephritis |
| 1Z1J.00 | 95406 | Chronic kidney disease stage 4 without proteinuria |
| 1Z1H.00 | 95122 | Chronic kidney disease stage 4 with proteinuria |
| K01..00 | 2999 | Nephrotic syndrome |
| 1Z12.00 | 12566 | Chronic kidney disease stage 3 |
| K02z.00 | 15097 | Chronic glomerulonephritis NOS |
| K022.00 | 61494 | Chronic membranoproliferative glomerulonephritis |
| 1Z1L.00 | 95405 | Chronic kidney disease stage 5 without proteinuria |
| K100100 | 57568 | Chronic pyelonephritis with medullary necrosis |
| K01B.00 | 17365 | Nephrotic syndrome, diffuse crescentic glomerulonephritis |
| K02..00 | 7804 | Chronic glomerulonephritis |
| 1Z1G.00 | 95177 | Chronic kidney disease stage 3B without proteinuria |
| K015.00 | 22852 | Nephrotic syndrome, focal and segmental glomerular lesions |
| K019.00 | 21989 | Nephrotic syn,diffuse mesangiocapillary glomerulonephritis |
| K01x111 | 45499 | Kimmelstiel - Wilson disease |
| 1Z16.00 | 95179 | Chronic kidney disease stage 3B |
| 1Z1E.00 | 95175 | Chronic kidney disease stage 3A without proteinuria |
| K01y.00 | 94373 | Nephrotic syndrome with other pathological kidney lesions |
| K010.00 | 9840 | Nephrotic syndrome with proliferative glomerulonephritis |
| K100z00 | 48111 | Chronic pyelonephritis NOS |
| K05..11 | 10081 | Chronic uraemia |
| K023.00 | 65064 | Chronic rapidly progressive glomerulonephritis |
| K050.00 | 6712 | End stage renal failure |
| K011.00 | 1803 | Nephrotic syndrome with membranous glomerulonephritis |
| 1Z1F.00 | 95178 | Chronic kidney disease stage 3B with proteinuria |
| 1Z1K.00 | 95508 | Chronic kidney disease stage 5 with proteinuria |
| K100.00 | 5531 | Chronic pyelonephritis |
| 1Z1C.00 | 95123 | Chronic kidney disease stage 3 without proteinuria |
| K02y.00 | 60960 | Other chronic glomerulonephritis |
| 1Z13.00 | 12479 | Chronic kidney disease stage 4 |
| 1Z1B.00 | 94793 | Chronic kidney disease stage 3 with proteinuria |
| K02yz00 | 63615 | Other chronic glomerulonephritis NOS |
| K01A.00 | 56987 | Nephrotic syndrome, dense deposit disease |
| K100300 | 25055 | Chronic pyonephrosis |
| K0D..00 | 8330 | End-stage renal disease |
| K013.00 | 29634 | Nephrotic syndrome with minimal change glomerulonephritis |
| K100200 | 21158 | Chronic pyelitis |
| K014.00 | 23913 | Nephrotic syndrome, minor glomerular abnormality |
| K016.00 | 19316 | Nephrotic syndrome, diffuse membranous glomerulonephritis |
| K100000 | 99631 | Chronic pyelonephritis without medullary necrosis |
| K012.00 | 99644 | Nephrotic syndrome+membranoproliferative glomerulonephritis |
| K01z.00 | 27427 | Nephrotic syndrome NOS |
| 1Z1D.00 | 95408 | Chronic kidney disease stage 3A with proteinuria |
| K02y000 | 97758 | Chronic glomerulonephritis + diseases EC |

Source: Kontopantelis et al (6)

## Depression codes

| **Read code** | **Med code** | **Description** |
| --- | --- | --- |
| Eu20400 | 20785 | [X]Post-schizophrenic depression |
| Eu33214 | 73991 | [X]Vital depression, recurrent without psychotic symptoms |
| E113700 | 6482 | Recurrent depression |
| Eu33y00 | 47731 | [X]Other recurrent depressive disorders |
| E118.00 | 10825 | Seasonal affective disorder |
| Eu32.13 | 7604 | [X]Single episode of reactive depression |
| E112.11 | 5879 | Agitated depression |
| E002100 | 21887 | Senile dementia with depression |
| Eu33.14 | 28756 | [X]Seasonal depressive disorder |
| E113300 | 25697 | Recurrent major depressive episodes, severe, no psychosis |
| Eu32000 | 11717 | [X]Mild depressive episode |
| E113z00 | 25563 | Recurrent major depressive episode NOS |
| Eu33z11 | 36616 | [X]Monopolar depression NOS |
| Eu33200 | 33469 | [X]Recurr depress disorder cur epi severe without psyc sympt |
| E112.14 | 595 | Endogenous depression |
| E112200 | 15155 | Single major depressive episode, moderate |
| E113100 | 29342 | Recurrent major depressive episodes, mild |
| E112400 | 32159 | Single major depressive episode, severe, with psychosis |
| Eu33211 | 11329 | [X]Endogenous depression without psychotic symptoms |
| E004300 | 43292 | Arteriosclerotic dementia with depression |
| E112000 | 34390 | Single major depressive episode, unspecified |
| Eu32800 | 98417 | [X]Major depression, severe with psychotic symptoms |
| Eu32311 | 24117 | [X]Single episode of major depression and psychotic symptoms |
| Eu32212 | 22806 | [X]Single episode major depression w'out psychotic symptoms |
| Eu33316 | 37764 | [X]Recurrent severe episodes/reactive depressive psychosis |
| E113.11 | 6932 | Endogenous depression - recurrent |
| Eu32400 | 10667 | [X]Mild depression |
| Eu32z00 | 2970 | [X]Depressive episode, unspecified |
| Eu33311 | 23731 | [X]Endogenous depression with psychotic symptoms |
| Eu33315 | 16861 | [X]Recurrent severe episodes of psychotic depression |
| E113600 | 55384 | Recurrent major depressive episodes, in full remission |
| 9Ov..00 | 51258 | Depression monitoring administration |
| 9H90.00 | 12399 | Depression annual review |
| E11z200 | 9183 | Masked depression |
| Eu33300 | 47009 | [X]Recurrent depress disorder cur epi severe with psyc symp |
| Eu32z13 | 28248 | [X]Prolonged single episode of reactive depression |
| Eu34112 | 10290 | [X]Depressive personality disorder |
| Eu32500 | 98346 | [X]Major depression, mild |
| E113.00 | 15099 | Recurrent major depressive episode |
| E113000 | 35671 | Recurrent major depressive episodes, unspecified |
| Eu32314 | 28863 | [X]Single episode of reactive depressive psychosis |
| Eu32700 | 98414 | [X]Major depression, severe without psychotic symptoms |
| E112.00 | 10610 | Single major depressive episode |
| Eu33z00 | 44300 | [X]Recurrent depressive disorder, unspecified |
| Eu32600 | 98252 | [X]Major depression, moderately severe |
| E112300 | 15219 | Single major depressive episode, severe, without psychosis |
| Eu32.00 | 4639 | [X]Depressive episode |
| E112.13 | 6950 | Endogenous depression first episode |
| Eu32313 | 24112 | [X]Single episode of psychotic depression |
| Eu33212 | 11252 | [X]Major depression, recurrent without psychotic symptoms |
| Eu32.12 | 18510 | [X]Single episode of psychogenic depression |
| E001300 | 27677 | Presenile dementia with depression |
| Eu32300 | 12099 | [X]Severe depressive episode with psychotic symptoms |
| E11..12 | 2560 | Depressive psychoses |
| Eu32z11 | 543 | [X]Depression NOS |
| E113500 | 56273 | Recurrent major depressive episodes,partial/unspec remission |
| Eu32.11 | 9055 | [X]Single episode of depressive reaction |
| Eu33400 | 22116 | [X]Recurrent depressive disorder, currently in remission |
| E130.00 | 8478 | Reactive depressive psychosis |
| Eu32y12 | 56609 | [X]Single episode of masked depression NOS |
| E113400 | 24171 | Recurrent major depressive episodes, severe, with psychosis |
| E112500 | 43324 | Single major depressive episode, partial or unspec remission |
| Eu33.13 | 8902 | [X]Recurrent episodes of reactive depression |
| Eu33314 | 31757 | [X]Recurr severe episodes/psychogenic depressive psychosis |
| E112z00 | 7011 | Single major depressive episode NOS |
| E112100 | 16506 | Single major depressive episode, mild |
| E2B..00 | 324 | Depressive disorder NEC |
| E291.00 | 16632 | Prolonged depressive reaction |
| Eu33.11 | 8851 | [X]Recurrent episodes of depressive reaction |
| Eu33100 | 29520 | [X]Recurrent depressive disorder, current episode moderate |
| Eu32211 | 41989 | [X]Single episode agitated depressn w'out psychotic symptoms |
| Eu32213 | 59386 | [X]Single episode vital depression w'out psychotic symptoms |
| E130.11 | 17770 | Psychotic reactive depression |
| E113200 | 14709 | Recurrent major depressive episodes, moderate |
| E112.12 | 6546 | Endogenous depression first episode |
| Eu33313 | 32941 | [X]Recurr severe episodes/major depression+psychotic symptom |
| Eu33.12 | 19696 | [X]Recurrent episodes of psychogenic depression |
| Eu33213 | 29451 | [X]Manic-depress psychosis,depressd,no psychotic symptoms |
| Eu34113 | 7737 | [X]Neurotic depression |
| Eu33.15 | 8826 | [X]SAD - Seasonal affective disorder |
| Eu33.00 | 3292 | [X]Recurrent depressive disorder |
| E135.00 | 1055 | Agitated depression |
| Eu34111 | 8584 | [X]Depressive neurosis |
| Eu32y00 | 6854 | [X]Other depressive episodes |
| Eu32100 | 9211 | [X]Moderate depressive episode |
| Eu32y11 | 10720 | [X]Atypical depression |
| Eu32z12 | 3291 | [X]Depressive disorder NOS |
| Eu33000 | 29784 | [X]Recurrent depressive disorder, current episode mild |
| Eu32z14 | 5987 | [X] Reactive depression NOS |
| Eu32200 | 9667 | [X]Severe depressive episode without psychotic symptoms |
| E11y200 | 27491 | Atypical depressive disorder |
| E2B1.00 | 4323 | Chronic depression |

Source: Kuan et al (3)

## Anxiety codes

| **Read code** | **Med code** | **Description** |
| --- | --- | --- |
| 1B1H.11 | 5347 | Fear |
| 1Bb..00 | 18672 | Specific fear |
| 225J.00 | 19000 | O/E - panic attack |
| 225K.00 | 26331 | O/E - fearful mood |
| E200.00 | 636 | Anxiety states |
| E200000 | 6939 | Anxiety state unspecified |
| E200100 | 4069 | Panic disorder |
| E200111 | 462 | Panic attack |
| E200200 | 4659 | Generalised anxiety disorder |
| E200300 | 655 | Anxiety with depression |
| E200400 | 1758 | Chronic anxiety |
| E200500 | 4634 | Recurrent anxiety |
| E200z00 | 4534 | Anxiety state NOS |
| E203.00 | 3208 | Obsessive-compulsive disorders |
| E203z00 | 15566 | Obsessive-compulsive disorder NOS |
| E292000 | 6221 | Separation anxiety disorder |
| Eu40.00 | 9386 | [X]Phobic anxiety disorders |
| Eu41.00 | 5385 | [X]Other anxiety disorders |
| Eu41000 | 8205 | [X]Panic disorder [episodic paroxysmal anxiety] |
| Eu41011 | 6408 | [X]Panic attack |
| Eu41012 | 4081 | [X]Panic state |
| Eu41100 | 10344 | [X]Generalized anxiety disorder |
| Eu41111 | 962 | [X]Anxiety neurosis |
| Eu41112 | 35825 | [X]Anxiety reaction |
| Eu41113 | 50191 | [X]Anxiety state |
| Eu41200 | 11913 | [X]Mixed anxiety and depressive disorder |
| Eu41211 | 7749 | [X]Mild anxiety depression |
| Eu41300 | 44321 | [X]Other mixed anxiety disorders |
| Eu41y00 | 24066 | [X]Other specified anxiety disorders |
| Eu41y11 | 28167 | [X]Anxiety hysteria |
| Eu41z00 | 23838 | [X]Anxiety disorder, unspecified |
| Eu41z11 | 25638 | [X]Anxiety NOS |
| Eu42.00 | 5304 | [X]Obsessive - compulsive disorder |
| Eu42y00 | 38809 | [X]Other obsessive-compulsive disorders |
| Eu42z00 | 22721 | [X]Obsessive-compulsive disorder, unspecified |

Source: Kuan et al (3)

## Insomnia codes

| **Read code** | **Med code** | **Description** |
| --- | --- | --- |
| 1B1B.00 | 21305 | Cannot sleep - insomnia |
| 1B1B000 | 3523 | Initial insomnia |
| 1B1B100 | 5675 | Middle insomnia |
| 1B1B.11 | 4537 | C/O - insomnia |
| 1B1B200 | 4597 | Late insomnia |
| 1BX0.00 | 42847 | Delayed onset of sleep |
| 1BX3.00 | 22465 | early morning waking |
| 1BX9.00 | 60974 | Light sleep |
| E274100 | 15515 | Transient insomnia |
| E274111 | 4023 | Insomnia NOS |
| E274.12 | 26546 | Insomnia due to nonorganic sleep disorder |
| E274200 | 16115 | Persistent insomnia |
| E274D11 | 19514 | Restless sleep |
| E274E00 | 32987 | 'Short-sleeper' |
| Eu51000 | 30626 | [X]Nonorganic insomnia |
| Fy00.00 | 5921 | Disorders of maintaining and initiating sleep |
| R005200 | 750 | [D]Insomnia NOS |

Source: Hoile et al (7)

## Antipsychotic medication codes

| **Prod code** | **BNF code** | **Description** |
| --- | --- | --- |
| 10107 | 0402010 | Quetiapine Starter Pack |
| 10405 | 0402010 | Thioridazine 25mg/5ml sugar free Oral solution |
| 10434 | 0402010 | Largactil 25mg/5ml Oral solution (Hawgreen Ltd) |
| 10435 | 0402010 | Haloperidol 10mg/ml Oral solution |
| 10514 | 0402010 | Fluphenazine decanoate 100mg/ml Injection |
| 10565 | 0402010 | Haloperidol decanoate 50mg/1ml solution for injection ampoules |
| 10666 | 0402010 | Dolmatil 200mg tablets (Sanofi) |
| 10780 | 0402010 | Promazine 50mg/5ml oral solution |
| 10944 | 0402010 | Pipotiazine palmitate 50mg/ml depot injection |
| 11213 | 0402010 | Haloperidol 2mg/5ml sugar free Oral solution |
| 11531 | 0402010 | Trifluoperazine 5mg/5ml oral solution sugar free |
| 11799 | 0402010 | Risperdal 6mg tablets (Janssen-Cilag Ltd) |
| 11821 | 0402010 | Risperdal Quicklet 500microgram orodispersible tablets (Janssen-Cilag Ltd) |
| 11828 | 0402010 | Risperidone 2mg orodispersible tablets sugar free |
| 1192 | 0402010 | Thioridazine 10mg tablets |
| 11938 | 0402010 | Amisulpride 25mg/5ml oral suspension |
| 12073 | 0402010 | Clopixol Conc 500mg/1ml solution for injection ampoules (Lundbeck Ltd) |
| 12128 | 0402010 | Modecate concentrate 100mg/ml Injection (Sanofi-Synthelabo Ltd) |
| 1218 | 0402010 | Thioridazine 25mg tablets |
| 12193 | 0402010 | Sparine 25mg Tablet (Wyeth Pharmaceuticals) |
| 12195 | 0402010 | Pericyazine 10mg/5ml oral solution |
| 12224 | 0402010 | Zuclopenthixol decanoate 500mg/1ml solution for injection ampoules |
| 12340 | 0402010 | Piportil 50mg/ml Depot injection (JHC Healthcare Ltd) |
| 12386 | 0402010 | Haldol decanoate 100mg/1ml solution for injection ampoules (Janssen-Cilag Ltd) |
| 12387 | 0402010 | Haloperidol 20mg tablets |
| 12445 | 0402010 | Remoxipride 300mg capsule |
| 1245 | 0402010 | Trifluoperazine 5mg tablets |
| 1249 | 0402010 | Olanzapine 10mg tablets |
| 12666 | 0402010 | Sertindole 4mg tablets |
| 12707 | 0402010 | Zuclopenthixol 2mg tablets |
| 12921 | 0402010 | Haldol 2mg/ml oral solution (Janssen-Cilag Ltd) |
| 13105 | 0402010 | Haloperidol 2mg/ml Oral solution |
| 1314 | 0402010 | Thioridazine 50mg tablets |
| 13145 | 0402010 | Trifluoperazine 1mg/5ml oral solution sugar free |
| 1316 | 0402010 | Stelazine 5mg tablets (Mercury Pharma Group Ltd) |
| 1318 | 0402010 | Stelazine 1mg tablets (Mercury Pharma Group Ltd) |
| 1319 | 0402010 | Clopixol 10mg tablets (Lundbeck Ltd) |
| 1320 | 0402010 | Risperidone 1mg tablets |
| 1321 | 0402010 | Risperidone 2mg tablets |
| 13311 | 0402010 | Sparine 50mg/ml Injection (Wyeth Pharmaceuticals) |
| 13338 | 0402010 | Serenace 5mg tablets (Teva UK Ltd) |
| 13368 | 0402010 | Clopixol 2mg tablets (Lundbeck Ltd) |
| 13369 | 0402010 | Droleptan 1mg/ml Oral solution (Janssen-Cilag Ltd) |
| 13483 | 0402010 | Serenace 20mg tablets (Teva UK Ltd) |
| 13484 | 0402010 | Serenace 10mg tablets (Teva UK Ltd) |
| 13600 | 0402010 | Zuclopenthixol 25mg tablets |
| 13820 | 0402010 | Zyprexa 10mg tablets (Eli Lilly and Company Ltd) |
| 13888 | 0402010 | Zyprexa 10mg Velotabs (Eli Lilly and Company Ltd) |
| 13902 | 0402010 | Neulactil Forte syrup (Sanofi) |
| 14112 | 0402010 | Clozaril 100mg tablets (Novartis Pharmaceuticals UK Ltd) |
| 14130 | 0402010 | Depixol Low Volume 200mg/1ml solution for injection ampoules (Lundbeck Ltd) |
| 14344 | 0402010 | Aripiprazole 5mg tablets |
| 1453 | 0402010 | Triptafen m 2mg+10mg Tablet (Goldshield Pharmaceuticals Ltd) |
| 14576 | 0402010 | Zuclopenthixol acetate 50mg/ml oily injection |
| 14610 | 0402010 | Promazine 50mg/5ml oral solution sugar free |
| 14717 | 0402010 | Zyprexa 5mg Velotabs (Eli Lilly and Company Ltd) |
| 14767 | 0402010 | Risperdal Consta 50mg powder and solvent for suspension for injection vials (Janssen-Cilag Ltd) |
| 14789 | 0402010 | Risperdal Consta 37.5mg powder and solvent for suspension for injection vials (Janssen-Cilag Ltd) |
| 14813 | 0402010 | Seroquel 150mg tablets (AstraZeneca UK Ltd) |
| 14839 | 0402010 | Flupentixol 200mg/1ml solution for injection ampoules |
| 14858 | 0402010 | Abilify 15mg tablets (Otsuka Pharmaceuticals (U.K.) Ltd) |
| 14859 | 0402010 | Seroquel 25mg tablets (AstraZeneca UK Ltd) |
| 14889 | 0402010 | Depixol Conc 100mg/1ml solution for injection ampoules (Lundbeck Ltd) |
| 14966 | 0402010 | Flupentixol 20mg/1ml solution for injection ampoules |
| 14987 | 0402010 | Perphenazine 2mg/5ml oral solution sugar free |
| 15047 | 0402010 | Orap 4mg tablets (Janssen-Cilag Ltd) |
| 15128 | 0402010 | Droperidol 1mg/ml liquid |
| 15161 | 0402010 | Promazine 50mg/ml injection |
| 15171 | 0402010 | Droleptan 10mg Tablet (Janssen-Cilag Ltd) |
| 15395 | 0402010 | Promazine 12.5mg/5ml oral solution |
| 15418 | 0402010 | Largactil forte 100mg/5ml Oral suspension (Hawgreen Ltd) |
| 15472 | 0402010 | Pericyazine 25mg tablet |
| 15598 | 0402010 | Thioridazine 100mg/5ml sugar free Oral solution |
| 15814 | 0402010 | Haloperidol decanoate 100mg/1ml solution for injection ampoules |
| 16006 | 0402010 | Risperdal Quicklet 2mg orodispersible tablets (Janssen-Cilag Ltd) |
| 16103 | 0402010 | Olanzapine 20mg Orodispersible tablet |
| 16223 | 0402010 | Roxiam 300mg Capsule (AstraZeneca UK Ltd) |
| 16323 | 0402010 | Perphenazine 2mg with Amitriptyline 10mg tablet |
| 16407 | 0402010 | Zyprexa 15mg Velotabs (Eli Lilly and Company Ltd) |
| 16425 | 0402010 | Risperidone 37.5mg powder and solvent for suspension for injection vials |
| 16434 | 0402010 | Risperidone 25mg powder and solvent for suspension for injection vials |
| 16489 | 0402010 | Risperidone 50mg powder and solvent for suspension for injection vials |
| 16561 | 0402010 | Aripiprazole 30mg tablets |
| 16575 | 0402010 | Aripiprazole 1mg/ml oral solution |
| 16768 | 0402010 | Solian 50 tablets (Sanofi) |
| 16908 | 0402010 | Risperdal Consta 25mg powder and solvent for suspension for injection vials (Janssen-Cilag Ltd) |
| 16986 | 0402010 | Risperdal Quicklet 1mg orodispersible tablets (Janssen-Cilag Ltd) |
| 16998 | 0402010 | Sertindole 12mg tablets |
| 17050 | 0402010 | Sertindole 20mg tablets |
| 17087 | 0402010 | Perphenazine 5mg/ml injection |
| 17190 | 0402010 | Fluphenazine enanthate 25mg/ml Injection |
| 17227 | 0402010 | Chloractil 25mg Tablet (DDSA Pharmaceuticals Ltd) |
| 1733 | 0402010 | Flupentixol decanoate 20mg/ml Injection |
| 17379 | 0402010 | Haloperidol 1.5mg/5ml sugar free Oral solution |
| 17399 | 0402010 | Thioridazine 50mg/5ml Oral solution |
| 17504 | 0402010 | Zotepine 25mg tablets |
| 17634 | 0402010 | Promazine 50mg/5ml oral solution |
| 17958 | 0402010 | Clozaril 25mg tablets (Novartis Pharmaceuticals UK Ltd) |
| 18013 | 0402010 | Seroquel 100mg tablets (AstraZeneca UK Ltd) |
| 18024 | 0402010 | Zyprexa 5mg tablets (Eli Lilly and Company Ltd) |
| 18132 | 0402010 | Abilify 5mg tablets (Otsuka Pharmaceuticals (U.K.) Ltd) |
| 18155 | 0402010 | Flupentixol 50mg/0.5ml solution for injection ampoules |
| 18175 | 0402010 | Flupentixol 100mg/1ml solution for injection ampoules |
| 18181 | 0402010 | Sulpor 200mg/5ml oral solution (Rosemont Pharmaceuticals Ltd) |
| 18197 | 0402010 | Depixol Conc 50mg/0.5ml solution for injection ampoules (Lundbeck Ltd) |
| 18289 | 0402010 | Stelazine 10mg/ml Concentrate (Goldshield Pharmaceuticals Ltd) |
| 18352 | 0402010 | Sulpitil 200mg tablets (Pfizer Ltd) |
| 18453 | 0402010 | Zyprexa 2.5mg tablets (Eli Lilly and Company Ltd) |
| 1857 | 0402010 | Trifluoperazine 1mg tablets |
| 18668 | 0402010 | Trifluoperazine 10mg/ml concentrate |
| 19002 | 0402010 | Largactil 100mg Suppository (Rhone-Poulenc Rorer Ltd) |
| 19016 | 0402010 | Roxiam 150mg Capsule (AstraZeneca UK Ltd) |
| 19283 | 0402010 | Depixol 20mg/1ml solution for injection ampoules (Lundbeck Ltd) |
| 19900 | 0402010 | Sertindole 16mg tablets |
| 19976 | 0402010 | Zyprexa 15mg tablets (Eli Lilly and Company Ltd) |
| 2094 | 0402010 | Haldol decanoate 50mg/1ml solution for injection ampoules (Janssen-Cilag Ltd) |
| 21027 | 0402010 | Triperidol 1mg Tablet (Lagap) |
| 21047 | 0402010 | Triperidol 0.5mg Tablet (Lagap) |
| 21064 | 0402010 | Neulactil 25mg Tablet (JHC Healthcare Ltd) |
| 21125 | 0402010 | Droleptan 5mg/ml Injection (Janssen-Cilag Ltd) |
| 21199 | 0402010 | Denzapine 100mg tablets (Britannia Pharmaceuticals Ltd) |
| 21339 | 0402010 | Veractil 25mg Tablet (Rhone-Poulenc Rorer Ltd) |
| 2135 | 0402010 | Sulpiride 200mg tablets |
| 2136 | 0402010 | Depixol 20mg/ml Injection (Lundbeck Ltd) |
| 2154 | 0402010 | Chlorpromazine 100mg tablets |
| 2155 | 0402010 | Depixol -conc 100mg/ml Injection (Lundbeck Ltd) |
| 2156 | 0402010 | Depixol 40mg/2ml solution for injection ampoules (Lundbeck Ltd) |
| 2157 | 0402010 | Perphenazine 4mg tablets |
| 21709 | 0402010 | Seroquel 300mg tablets (AstraZeneca UK Ltd) |
| 21744 | 0402010 | Anquil 250microgram Tablet (Concord Pharmaceuticals Ltd) |
| 21964 | 0402010 | Zyprexa 7.5mg tablets (Eli Lilly and Company Ltd) |
| 22049 | 0402010 | Clopixol 200mg/1ml solution for injection ampoules (Lundbeck Ltd) |
| 22606 | 0402010 | Chlorpromazine 25mg/1ml solution for injection ampoules |
| 22609 | 0402010 | Droperidol 5mg/ml injection |
| 22660 | 0402010 | Haldol 5mg tablets (Janssen-Cilag Ltd) |
| 2276 | 0402010 | Flupentixol 40mg/2ml solution for injection ampoules |
| 228 | 0402010 | Fentazin 5mg/ml Injection (Goldshield Pharmaceuticals Ltd) |
| 22814 | 0402010 | Trifluperidol 1mg Tablet |
| 23034 | 0402010 | Remoxipride 75mg capsule |
| 23162 | 0402010 | Serdolect 16mg tablets (Lundbeck Ltd) |
| 23431 | 0402010 | Olanzapine 10mg powder for solution for injection vials |
| 23659 | 0402010 | Trifluperidol 0.5mg Tablet |
| 23678 | 0402010 | Haldol 10mg tablets (Janssen-Cilag Ltd) |
| 24053 | 0402010 | Sulparex 200mg Tablet (E R Squibb and Sons Ltd) |
| 24069 | 0402010 | Dolmatil 400mg tablets (Sanofi) |
| 2419 | 0402010 | Haloperidol 500microgram capsules |
| 24270 | 0402010 | Zuclopenthixol acetate 50mg/1ml solution for injection ampoules |
| 24358 | 0402010 | Abilify 10mg tablets (Otsuka Pharmaceuticals (U.K.) Ltd) |
| 24494 | 0402010 | Haldol 10mg/ml Liquid (Janssen-Cilag Ltd) |
| 24890 | 0402010 | Trifluoperazine with tranylcypromine 1mg + 10mg Tablet |
| 25336 | 0402010 | Zotepine 50mg tablets |
| 2540 | 0402010 | Benperidol 250microgram tablets |
| 25653 | 0402010 | Chloractil 50mg Tablet (DDSA Pharmaceuticals Ltd) |
| 25835 | 0402010 | Moditen enanthate 25mg/ml Injection (Sanofi-Synthelabo Ltd) |
| 25909 | 0402010 | Perphenazine 4mg/5ml Oral solution sugar free |
| 25966 | 0402010 | Serdolect 4mg tablets (Lundbeck Ltd) |
| 2620 | 0402010 | Haloperidol 1mg/ml Oral solution |
| 2621 | 0402010 | Haloperidol 5mg tablets |
| 26544 | 0402010 | Solian 100 tablets (Sanofi) |
| 2656 | 0402010 | Olanzapine 2.5mg tablets |
| 27148 | 0402010 | Orap 10mg Tablet (Janssen-Cilag Ltd) |
| 27211 | 0402010 | Integrin 40mg Tablet (Sanofi-Synthelabo Ltd) |
| 2786 | 0402010 | Risperidone 6mg tablets |
| 2787 | 0402010 | Risperidone 4mg tablets |
| 2801 | 0402010 | Thioridazine 10mg/5ml Oral solution |
| 2814 | 0402010 | Largactil 25mg Tablet (Hawgreen Ltd) |
| 28147 | 0402010 | Taractan 15mg Tablet (Roche Products Ltd) |
| 28231 | 0402010 | Levinan 6mg Tablet (Link Pharmaceuticals Ltd) |
| 28355 | 0402010 | Zuclopenthixol decanoate 200mg/1ml solution for injection ampoules |
| 28679 | 0402010 | Dozic 2mg/ml Oral solution (Rosemont Pharmaceuticals Ltd) |
| 28759 | 0402010 | Zotepine 100mg tablets |
| 28862 | 0402010 | Chloractil 100mg Tablet (DDSA Pharmaceuticals Ltd) |
| 29540 | 0402010 | Olanzapine 20mg tablets |
| 2972 | 0402010 | Promazine 25mg tablets |
| 29879 | 0402010 | Abilify 30mg tablets (Otsuka Pharmaceuticals (U.K.) Ltd) |
| 29948 | 0402010 | Stelazine Forte 1mg/ml oral solution (Mercury Pharma Group Ltd) |
| 30088 | 0402010 | Zoleptil 25 tablets (Movianto UK Ltd) |
| 30111 | 0402010 | Chlorprothixene 50mg tablets |
| 302 | 0402010 | Risperidone 1mg/ml oral solution sugar free |
| 3021 | 0402010 | Thioridazine 100mg tablets |
| 30487 | 0402010 | Denzapine 25mg tablets (Britannia Pharmaceuticals Ltd) |
| 31063 | 0402010 | Serdolect 12mg tablets (Lundbeck Ltd) |
| 31098 | 0402010 | Aripiprazole 15mg orodispersible tablets sugar free |
| 31171 | 0402010 | Chlorpromazine 50mg tablets (A A H Pharmaceuticals Ltd) |
| 31172 | 0402010 | Chlorpromazine 50mg tablets (Teva UK Ltd) |
| 31175 | 0402010 | Chlorpromazine 25mg tablets (A A H Pharmaceuticals Ltd) |
| 31184 | 0402010 | Chlorpromazine 25mg tablets (IVAX Pharmaceuticals UK Ltd) |
| 31537 | 0402010 | Zuclopenthixol acetate 100mg/2ml solution for injection ampoules |
| 31538 | 0402010 | Clopixol Acuphase 100mg/2ml solution for injection ampoules (Lundbeck Ltd) |
| 31576 | 0402010 | Solian 100mg/ml oral solution (Sanofi) |
| 31796 | 0402010 | Benquil 250microgram tablets (Concord Pharmaceuticals Ltd) |
| 3197 | 0402010 | Promazine 100mg tablet |
| 32051 | 0402010 | Haloperidol 5mg Tablet (Generics (UK) Ltd) |
| 32076 | 0402010 | Aripiprazole 10mg orodispersible tablets sugar free |
| 3226 | 0402010 | Sparine 50mg Tablet (Wyeth Pharmaceuticals) |
| 3227 | 0402010 | Sparine 50mg/5ml Liquid (Wyeth Pharmaceuticals) |
| 3228 | 0402010 | Promazine 50mg tablets |
| 3233 | 0402010 | Haloperidol 2mg/ml sugar free Liquid |
| 3281 | 0402010 | Olanzapine 5mg tablets |
| 32838 | 0402010 | Haloperidol 1.5mg tablets (IVAX Pharmaceuticals UK Ltd) |
| 329 | 0402010 | Haloperidol 1.5mg tablets |
| 3348 | 0402010 | Chlorpromazine 50mg tablets |
| 33493 | 0402010 | Sparine 100mg Tablet (Wyeth Pharmaceuticals) |
| 3356 | 0402010 | Parstelin Tablet (GlaxoSmithKline Consumer Healthcare) |
| 33780 | 0402010 | Modecate 25mg/1ml solution for injection ampoules (Sanofi) |
| 33883 | 0402010 | Zyprexa 20mg Velotabs (Eli Lilly and Company Ltd) |
| 34039 | 0402010 | Haloperidol 1mg/ml Liquid (Rosemont Pharmaceuticals Ltd) |
| 34272 | 0402010 | Haloperidol 5mg/ml Injection (Antigen Pharmaceuticals) |
| 34339 | 0402010 | Haloperidol 1.5mg tablets (A A H Pharmaceuticals Ltd) |
| 34630 | 0402010 | Chlorpromazine 50mg tablets (Thornton & Ross Ltd) |
| 34668 | 0402010 | Chlorpromazine 25mg tablets (Teva UK Ltd) |
| 34693 | 0402010 | Chlorpromazine 25mg tablets (Thornton & Ross Ltd) |
| 34736 | 0402010 | Chlorpromazine 100mg tablets (Teva UK Ltd) |
| 34810 | 0402010 | Sulpiride 200mg tablets (Wockhardt UK Ltd) |
| 34903 | 0402010 | Haloperidol 5mg tablets (IVAX Pharmaceuticals UK Ltd) |
| 34905 | 0402010 | Thioridazine 25mg Tablet (IVAX Pharmaceuticals UK Ltd) |
| 34927 | 0402010 | Amisulpride 200mg tablets (Zentiva) |
| 35065 | 0402010 | Fluphenazine decanoate 25mg/1ml solution for injection ampoules |
| 35122 | 0402010 | Modecate 12.5mg/0.5ml solution for injection ampoules (Sanofi) |
| 35141 | 0402010 | Risperidone 3mg orodispersible tablets sugar free |
| 35176 | 0402010 | Fluphenazine decanoate 100mg/1ml solution for injection ampoules |
| 35235 | 0402010 | Piportil Depot 50mg/1ml solution for injection ampoules (Sanofi) |
| 35391 | 0402010 | Fluphenazine decanoate 50mg/0.5ml solution for injection ampoules |
| 35445 | 0402010 | Modecate 50mg/2ml solution for injection ampoules (Sanofi) |
| 35455 | 0402010 | Modecate Concentrate 100mg/1ml solution for injection ampoules (Sanofi) |
| 35487 | 0402010 | Modecate Concentrate 50mg/0.5ml solution for injection ampoules (Sanofi) |
| 35488 | 0402010 | Piportil Depot 100mg/2ml solution for injection ampoules (Sanofi) |
| 35530 | 0402010 | Fluphenazine decanoate 12.5mg/0.5ml solution for injection ampoules |
| 35548 | 0402010 | Risperdal Quicklet 3mg orodispersible tablets (Janssen-Cilag Ltd) |
| 35589 | 0402010 | Risperidone 4mg orodispersible tablets sugar free |
| 35684 | 0402010 | Pipotiazine 50mg/1ml solution for injection ampoules |
| 35723 | 0402010 | Fluphenazine decanoate 50mg/2ml solution for injection ampoules |
| 35787 | 0402010 | Thioridazine 50mg Tablet (IVAX Pharmaceuticals UK Ltd) |
| 35929 | 0402010 | Chlorpromazine 50mg/2ml solution for injection ampoules |
| 35953 | 0402010 | Risperdal Quicklet 4mg orodispersible tablets (Janssen-Cilag Ltd) |
| 3605 | 0402010 | Thioridazine 25mg/5ml oral solution |
| 36101 | 0402010 | Clopixol Acuphase 50mg/1ml solution for injection ampoules (Lundbeck Ltd) |
| 36116 | 0402010 | Paliperidone 6mg modified-release tablets |
| 36163 | 0402010 | Zyprexa 20mg tablets (Eli Lilly and Company Ltd) |
| 36394 | 0402010 | Pipotiazine 100mg/2ml solution for injection ampoules |
| 36771 | 0402010 | Haloperidol 250micrograms/5ml oral suspension |
| 36954 | 0402010 | Invega 6mg modified-release tablets (Janssen-Cilag Ltd) |
| 37501 | 0402010 | Paliperidone 9mg modified-release tablets |
| 37606 | 0402010 | Abilify 10mg orodispersible tablets (Otsuka Pharmaceuticals (U.K.) Ltd) |
| 37705 | 0402010 | Chlorpromazine 100mg/5ml suspension |
| 37717 | 0402010 | Paliperidone 3mg modified-release tablets |
| 3772 | 0402010 | Largactil 50mg Tablet (Hawgreen Ltd) |
| 3773 | 0402010 | Droperidol 10mg tablets |
| 3774 | 0402010 | Clopixol 200mg/ml Oily injection (Lundbeck Ltd) |
| 3775 | 0402010 | Zuclopenthixol decanoate 200mg/ml oily injection |
| 37871 | 0402010 | Chlorpromazine 25mg/5ml Oral solution (Rosemont Pharmaceuticals Ltd) |
| 38010 | 0402010 | Abilify 15mg orodispersible tablets (Otsuka Pharmaceuticals (U.K.) Ltd) |
| 38080 | 0402010 | Abilify 1mg/ml oral solution (Otsuka Pharmaceuticals (U.K.) Ltd) |
| 38089 | 0402010 | Promazine 50mg/5ml syrup (Rosemont Pharmaceuticals Ltd) |
| 38262 | 0402010 | Haloperidol 5mg/1ml solution for injection ampoules |
| 38375 | 0402010 | Aripiprazole 9.75mg/1.3ml solution for injection vials |
| 38540 | 0402010 | Haldol 5mg/1ml solution for injection ampoules (Janssen-Cilag Ltd) |
| 38840 | 0402010 | Quetiapine 400mg modified-release tablets |
| 38885 | 0402010 | Quetiapine 50mg modified-release tablets |
| 38906 | 0402010 | Quetiapine 300mg modified-release tablets |
| 38912 | 0402010 | Quetiapine 200mg modified-release tablets |
| 38913 | 0402010 | Seroquel XL 50mg tablets (AstraZeneca UK Ltd) |
| 38914 | 0402010 | Seroquel XL 200mg tablets (AstraZeneca UK Ltd) |
| 38937 | 0402010 | Seroquel XL 300mg tablets (AstraZeneca UK Ltd) |
| 39237 | 0402010 | Seroquel XL 400mg tablets (AstraZeneca UK Ltd) |
| 3926 | 0402010 | Modecate 25mg/ml Injection (Sanofi-Synthelabo Ltd) |
| 3952 | 0402010 | Chlorpromazine 25mg/5ml oral solution |
| 3955 | 0402010 | Tranylcypromine with trifluoperazine Tablet |
| 39830 | 0402010 | Neulactil 2.5mg tablets (Sanofi) |
| 40162 | 0402010 | Trifluoperazine 1mg tablets (A A H Pharmaceuticals Ltd) |
| 40390 | 0402010 | Promazine 25mg/5ml syrup (Rosemont Pharmaceuticals Ltd) |
| 40586 | 0402010 | Clozapine 50mg tablets |
| 40587 | 0402010 | Clozapine 200mg tablets |
| 40779 | 0402010 | Quetiapine 100mg/5ml oral solution |
| 40782 | 0402010 | Levomepromazine 6mg Tablet |
| 40881 | 0402010 | Neulactil 10mg tablets (Sanofi) |
| 40932 | 0402010 | Quetiapine 100mg/5ml oral suspension |
| 41070 | 0402010 | Denzapine 50mg tablets (Britannia Pharmaceuticals Ltd) |
| 41428 | 0402010 | Denzapine 200mg tablets (Britannia Pharmaceuticals Ltd) |
| 41546 | 0402010 | Haloperidol 1mg/ml Liquid (Hillcross Pharmaceuticals Ltd) |
| 41645 | 0402010 | Chlorpromazine 25mg/ml Injection (Antigen Pharmaceuticals) |
| 41663 | 0402010 | Trifluoperazine 5mg tablets (A A H Pharmaceuticals Ltd) |
| 41675 | 0402010 | Sulpiride 200mg tablets (IVAX Pharmaceuticals UK Ltd) |
| 41702 | 0402010 | Amisulpride 100mg tablets (Zentiva) |
| 41714 | 0402010 | Amisulpride 50mg tablets (Zentiva) |
| 41732 | 0402010 | Promazine 50mg tablets (Teva UK Ltd) |
| 41970 | 0402010 | Fluphenazine decanoate 25mg/1ml solution for injection ampoules (Hospira UK Ltd) |
| 41971 | 0402010 | Fluphenazine decanoate 25mg/ml Injection (Antigen Pharmaceuticals) |
| 41995 | 0402010 | Promazine 50mg/ml Injection (Genus Pharmaceuticals Ltd) |
| 42000 | 0402010 | Haloperidol 2mg/ml Liquid (Rosemont Pharmaceuticals Ltd) |
| 42229 | 0402010 | Droperidol oral liquid |
| 42242 | 0402010 | Clozapine 50mg/ml oral suspension sugar free |
| 4232 | 0402010 | Nozinan 25mg tablets (Sanofi) |
| 4234 | 0402010 | Haloperidol 5mg/ml Injection |
| 42807 | 0402010 | Haloperidol 500microgram Tablet (Lagap) |
| 42816 | 0402010 | Thioridazine 50mg/5ml Oral solution (Rosemont Pharmaceuticals Ltd) |
| 42895 | 0402010 | Haloperidol 5mg tablets (Teva UK Ltd) |
| 43020 | 0402010 | Haloperidol Oral solution |
| 43423 | 0402010 | Sulpiride 200mg tablets (A A H Pharmaceuticals Ltd) |
| 43520 | 0402010 | Haloperidol 1.5mg tablets (Teva UK Ltd) |
| 43522 | 0402010 | Sulpiride 200mg tablets (Teva UK Ltd) |
| 43654 | 0402010 | Promazine 50mg/ml injection |
| 43914 | 0402010 | Olanzapine embonate 210mg powder and solvent for suspension for injection vials |
| 44024 | 0402010 | Quetiapine 150mg modified-release tablets |
| 44186 | 0402010 | Chlorpromazine 25mg/5ml oral solution (A A H Pharmaceuticals Ltd) |
| 44326 | 0402010 | Seroquel XL 150mg tablets (AstraZeneca UK Ltd) |
| 4434 | 0402010 | Chlorpromazine 50mg/5ml oral solution |
| 4442 | 0402010 | Nozinan 25mg/1ml solution for injection ampoules (Sanofi) |
| 45281 | 0402010 | Chlorpromazine 100mg/5ml oral solution (Rosemont Pharmaceuticals Ltd) |
| 45444 | 0402010 | Denzapine 50mg/ml oral suspension (Britannia Pharmaceuticals Ltd) |
| 45810 | 0402010 | Haloperidol 10mg/5ml oral solution sugar free |
| 45839 | 0402010 | Quetiapine 25mg/5ml oral suspension |
| 45860 | 0402010 | Thioridazine 100mg Tablet (IVAX Pharmaceuticals UK Ltd) |
| 45880 | 0402010 | Haloperidol 5mg/5ml oral solution sugar free |
| 45953 | 0402010 | Zyprexa 10mg powder for solution for injection vials (Eli Lilly and Company Ltd) |
| 46224 | 0402010 | Paliperidone 50mg/0.5ml suspension for injection pre-filled syringes |
| 46351 | 0402010 | Paliperidone 150mg/1.5ml suspension for injection pre-filled syringes |
| 46422 | 0402010 | Olanzapine embonate 300mg powder and solvent for suspension for injection vials |
| 46434 | 0402010 | Xeplion 100mg/1ml suspension for injection pre-filled syringes (Janssen-Cilag Ltd) |
| 46435 | 0402010 | Xeplion 150mg/1.5ml suspension for injection pre-filled syringes (Janssen-Cilag Ltd) |
| 46436 | 0402010 | Xeplion 75mg/0.75ml suspension for injection pre-filled syringes (Janssen-Cilag Ltd) |
| 46447 | 0402010 | Paliperidone 100mg/1ml suspension for injection pre-filled syringes |
| 46556 | 0402010 | Paliperidone 75mg/0.75ml suspension for injection pre-filled syringes |
| 46677 | 0402010 | Risperidone 500microgram tablets (Actavis UK Ltd) |
| 46705 | 0402010 | Abilify 9.75mg/1.3ml solution for injection vials (Otsuka Pharmaceuticals (U.K.) Ltd) |
| 46764 | 0402010 | Quetiapine 12.5mg/5ml oral solution |
| 46871 | 0402010 | Quetiapine 12.5mg/5ml oral suspension |
| 46889 | 0402010 | Amisulpride 25mg/5ml oral solution |
| 46945 | 0402010 | Promazine 25mg Tablet (Biorex Laboratories Ltd) |
| 46960 | 0402010 | Chlorpromazine 100mg tablets (IVAX Pharmaceuticals UK Ltd) |
| 46969 | 0402010 | Amisulpride 200mg tablets (A A H Pharmaceuticals Ltd) |
| 47013 | 0402010 | Haloperidol 1mg/5ml oral suspension |
| 47049 | 0402010 | Olanzapine 10mg orodispersible tablets |
| 47055 | 0402010 | Olanzapine 5mg orodispersible tablets |
| 47063 | 0402010 | Olanzapine 10mg orodispersible tablets sugar free |
| 47083 | 0402010 | Olanzapine 20mg orodispersible tablets |
| 47093 | 0402010 | Olanzapine 20mg orodispersible tablets sugar free |
| 47098 | 0402010 | Olanzapine 5mg orodispersible tablets sugar free |
| 47103 | 0402010 | Olanzapine 15mg orodispersible tablets sugar free |
| 47149 | 0402010 | Haloperidol 1mg/5ml oral solution |
| 47152 | 0402010 | Olanzapine 15mg orodispersible tablets |
| 47162 | 0402010 | Xeplion 50mg/0.5ml suspension for injection pre-filled syringes (Janssen-Cilag Ltd) |
| 47167 | 0402010 | Asenapine 10mg sublingual tablets sugar free |
| 47233 | 0402010 | Zaponex 25mg tablets (Teva UK Ltd) |
| 47256 | 0402010 | Olanzapine 5mg oral lyophilisates sugar free |
| 47280 | 0402010 | Asenapine 5mg sublingual tablets sugar free |
| 47302 | 0402010 | Zaponex 100mg tablets (Teva UK Ltd) |
| 47304 | 0402010 | Olanzapine 10mg oral lyophilisates sugar free |
| 47361 | 0402010 | Thioridazine 10mg/5ml Oral solution (Rosemont Pharmaceuticals Ltd) |
| 47365 | 0402010 | Anquil 250microgram tablets (Archimedes Pharma UK Ltd) |
| 47394 | 0402010 | Olanzapine 15mg oral lyophilisates sugar free |
| 47498 | 0402010 | Olanzapine 20mg oral lyophilisates sugar free |
| 475 | 0402010 | Haloperidol 10mg tablets |
| 47808 | 0402010 | Haloperidol 10mg/5ml oral solution sugar free (A A H Pharmaceuticals Ltd) |
| 47832 | 0402010 | Risperidone 500microgram tablets (A A H Pharmaceuticals Ltd) |
| 48077 | 0402010 | Roxiam ir 75mg Capsule (AstraZeneca UK Ltd) |
| 4820 | 0402010 | Risperdal 1mg tablets (Janssen-Cilag Ltd) |
| 4876 | 0402010 | Amisulpride 50mg tablets |
| 49207 | 0402010 | Haloperidol 2mg/5ml oral solution |
| 49606 | 0402010 | Levinan 6mg tablets (Archimedes Pharma UK Ltd) |
| 49696 | 0402010 | Quetiapine 25mg/5ml oral solution |
| 49699 | 0402010 | Abilify 5mg tablets (Sigma Pharmaceuticals Plc) |
| 4992 | 0402010 | Solian 200 tablets (Sanofi) |
| 5014 | 0402010 | Levomepromazine 25mg tablets |
| 50214 | 0402010 | Olanzapine 5mg orodispersible tablets |
| 5039 | 0402010 | Quetiapine 100mg tablets |
| 5040 | 0402010 | Quetiapine 150mg tablets |
| 5071 | 0402010 | Amisulpride 200mg tablets |
| 51178 | 0402010 | Quetiapine 50mg/5ml oral suspension |
| 51240 | 0402010 | Risperidone 125micrograms/5ml oral solution |
| 51444 | 0402010 | Risperdal Consta 50mg powder and solvent for suspension for injection vials (Waymade Healthcare Plc) |
| 51558 | 0402010 | Amisulpride 12.5mg/5ml oral suspension |
| 5192 | 0402010 | Haloperidol 1mg/5ml sugar free Oral solution |
| 52001 | 0402010 | Olanzapine 2.5mg tablets (Aspire Pharma Ltd) |
| 52050 | 0402010 | Haloperidol 1.5mg/5ml oral suspension |
| 52076 | 0402010 | Amisulpride 12.5mg/5ml oral solution |
| 5212 | 0402010 | Fluphenazine 1mg tablets |
| 5219 | 0402010 | Risperidone 3mg tablets |
| 5262 | 0402010 | Risperdal 1mg/ml liquid (Janssen-Cilag Ltd) |
| 5283 | 0402010 | Quetiapine 25mg tablets |
| 52846 | 0402010 | Nozinan 25mg/1ml solution for injection ampoules (Lexon (UK) Ltd) |
| 52940 | 0402010 | Sondate XL 50mg tablets (Teva UK Ltd) |
| 53552 | 0402010 | Quetiapine 25mg tablets (Zentiva) |
| 53556 | 0402010 | Olanzapine 10mg oral lyophilisates sugar free |
| 53634 | 0402010 | Droperidol capsules |
| 53649 | 0402010 | Haloperidol 2mg/5ml oral suspension |
| 53848 | 0402010 | Zalasta 5mg orodispersible tablets (Consilient Health Ltd) |
| 53951 | 0402010 | Levomepromazine 6.25mg/5ml oral solution |
| 54346 | 0402010 | Risperidone 1mg tablets (Teva UK Ltd) |
| 54483 | 0402010 | Sondate XL 300mg tablets (Teva UK Ltd) |
| 55011 | 0402010 | Largactil 25mg tablets (Sanofi) |
| 55268 | 0402010 | Zypadhera 300mg powder and solvent for suspension for injection vials (Eli Lilly and Company Ltd) |
| 5545 | 0402010 | Serenace 500microgram capsules (Teva UK Ltd) |
| 55620 | 0402010 | Flupentixol Liquid |
| 55622 | 0402010 | Olanzapine 10mg orodispersible tablets |
| 55625 | 0402010 | Amisulpride 50mg/5ml oral suspension |
| 55661 | 0402010 | Risperidone 1mg tablets (Kent Pharmaceuticals Ltd) |
| 55667 | 0402010 | Olanzapine 15mg tablets (Actavis UK Ltd) |
| 55848 | 0402010 | Haloperidol 5mg/1ml solution for injection ampoules (AMCo) |
| 55870 | 0402010 | Quetiapine oral liquid |
| 55871 | 0402010 | Haloperidol 2mg/ml Liquid (Hillcross Pharmaceuticals Ltd) |
| 55890 | 0402010 | Promazine 50mg/5ml Liquid (Rosemont Pharmaceuticals Ltd) |
| 5597 | 0402010 | Moditen 1mg tablets (Sanofi) |
| 56072 | 0402010 | Olanzapine 20mg orodispersible tablets |
| 56143 | 0402010 | Olanzapine 15mg orodispersible tablets |
| 56215 | 0402010 | Quetiapine 50mg/5ml oral solution |
| 56265 | 0402010 | Olanzapine 20mg oral lyophilisates sugar free |
| 56387 | 0402010 | Risperidone 1mg/ml oral solution sugar free (Alliance Healthcare (Distribution) Ltd) |
| 5653 | 0402010 | Olanzapine 7.5mg tablets |
| 56647 | 0402010 | Quetiapine 300mg tablets (Arrow Generics Ltd) |
| 56862 | 0402010 | Chlorpromazine 25mg/5ml syrup (Rosemont Pharmaceuticals Ltd) |
| 57034 | 0402010 | Sondate XL 200mg tablets (Teva UK Ltd) |
| 5707 | 0402010 | Flupentixol 3mg tablets |
| 57114 | 0402010 | Abilify 5mg tablets (Mawdsley-Brooks & Company Ltd) |
| 5712 | 0402010 | Depixol 3mg tablets (Lundbeck Ltd) |
| 57160 | 0402010 | Olanzapine 5mg oral lyophilisates sugar free |
| 57170 | 0402010 | Psytixol 100mg/1ml solution for injection ampoules (Generics (UK) Ltd) |
| 57217 | 0402010 | Risperidone 1mg tablets (Generics (UK) Ltd) |
| 57270 | 0402010 | Olanzapine 2.5mg/5ml oral suspension |
| 57412 | 0402010 | Sondate XL 400mg tablets (Teva UK Ltd) |
| 57550 | 0402010 | Largactil 25mg/5ml syrup (Sanofi) |
| 57612 | 0402010 | Seroquel XL 400mg tablets (Lexon (UK) Ltd) |
| 57613 | 0402010 | Seroquel XL 50mg tablets (Sigma Pharmaceuticals Plc) |
| 57616 | 0402010 | Olanzapine 20mg tablets (Teva UK Ltd) |
| 5762 | 0402010 | Clopixol acuphase 50mg/ml Oily injection (Lundbeck Ltd) |
| 57762 | 0402010 | Psytixol 40mg/2ml solution for injection ampoules (Generics (UK) Ltd) |
| 58067 | 0402010 | Quetiapine 125mg/5ml oral suspension |
| 58147 | 0402010 | Olanzapine 10mg tablets (Zentiva) |
| 5821 | 0402010 | Pimozide 4mg tablets |
| 58425 | 0402010 | Seroquel XL 50mg tablets (DE Pharmaceuticals) |
| 58492 | 0402010 | Chlorpromazine 100mg tablets (Waymade Healthcare Plc) |
| 58702 | 0402010 | Largactil 100mg tablets (Sanofi) |
| 58703 | 0402010 | Largactil 50mg tablets (Sanofi) |
| 588 | 0402010 | Chlorpromazine 25mg tablets |
| 58821 | 0402010 | Quetiapine 25mg tablets (Dr Reddy's Laboratories (UK) Ltd) |
| 58822 | 0402010 | Risperidone 4mg tablets (Almus Pharmaceuticals Ltd) |
| 58854 | 0402010 | Olanzapine 10mg tablets (Actavis UK Ltd) |
| 58935 | 0402010 | Tenprolide XL 400mg tablets (Actavis UK Ltd) |
| 58936 | 0402010 | Tenprolide XL 50mg tablets (Actavis UK Ltd) |
| 59143 | 0402010 | Olanzapine 2.5mg tablets (Teva UK Ltd) |
| 59215 | 0402010 | Quetiapine 150mg tablets (Ranbaxy (UK) Ltd) |
| 5927 | 0402010 | Amisulpride 400mg tablets |
| 59548 | 0402010 | Risperidone 2mg tablets (Alliance Healthcare (Distribution) Ltd) |
| 59816 | 0402010 | Psytixol 50mg/0.5ml solution for injection ampoules (Generics (UK) Ltd) |
| 59829 | 0402010 | Risperidone 3mg tablets (A A H Pharmaceuticals Ltd) |
| 59938 | 0402010 | Levomepromazine 25mg/1ml solution for injection ampoules (Wockhardt UK Ltd) |
| 6023 | 0402010 | Olanzapine 10mg Orodispersible tablet |
| 60250 | 0402010 | Levomepromazine 3mg/5ml oral solution |
| 60450 | 0402010 | Promazine 25mg tablets (A A H Pharmaceuticals Ltd) |
| 6064 | 0402010 | Levomepromazine 25mg/1ml solution for injection ampoules |
| 609 | 0402010 | Perphenazine 2mg tablets |
| 6109 | 0402010 | Solian 400 tablets (Sanofi) |
| 6134 | 0402010 | Dozic 5mg/5ml oral solution (Rosemont Pharmaceuticals Ltd) |
| 631 | 0402010 | Risperdal 500microgram tablets (Janssen-Cilag Ltd) |
| 6373 | 0402010 | Risperidone 1mg orodispersible tablets sugar free |
| 6412 | 0402010 | Olanzapine 5mg Orodispersible tablet |
| 6443 | 0402010 | Promazine 25mg/5ml oral solution |
| 6482 | 0402010 | Amisulpride 100mg/ml oral solution sugar free |
| 6523 | 0402010 | Haldol 5mg/ml Injection (Janssen-Cilag Ltd) |
| 6524 | 0402010 | Amisulpride 100mg tablets |
| 6561 | 0402010 | Aripiprazole 10mg tablets |
| 6573 | 0402010 | Aripiprazole 15mg tablets |
| 667 | 0402010 | Risperidone 500microgram tablets |
| 6838 | 0402010 | Olanzapine 15mg Orodispersible tablet |
| 6850 | 0402010 | Olanzapine 15mg tablets |
| 6864 | 0402010 | Seroquel 200mg tablets (AstraZeneca UK Ltd) |
| 6894 | 0402010 | Perphenazine 2mg with Amitriptyline 25mg tablet |
| 7039 | 0402010 | Quetiapine 300mg tablets |
| 7382 | 0402010 | Risperidone 500microgram orodispersible tablets sugar free |
| 7390 | 0402010 | Levomepromazine 6mg tablets |
| 7436 | 0402010 | Serenace 5mg/1ml solution for injection ampoules (IVAX Pharmaceuticals UK Ltd) |
| 7479 | 0402010 | Stelazine 1mg/ml Injection (Goldshield Pharmaceuticals Ltd) |
| 7493 | 0402010 | Largactil 100mg Tablet (Hawgreen Ltd) |
| 7514 | 0402010 | Largactil 50mg/2ml solution for injection ampoules (Sanofi) |
| 7833 | 0402010 | Neulactil 2.5mg Tablet (JHC Healthcare Ltd) |
| 7834 | 0402010 | Pericyazine 2.5mg tablets |
| 7919 | 0402010 | Fentazin 4mg tablets (AMCo) |
| 8031 | 0402010 | Neulactil 10mg Tablet (JHC Healthcare Ltd) |
| 8032 | 0402010 | Pericyazine 10mg tablets |
| 8046 | 0402010 | Clozapine 25mg tablets |
| 8047 | 0402010 | Clozapine 100mg tablets |
| 8153 | 0402010 | Serenace 2mg/ml liquid (Teva UK Ltd) |
| 8311 | 0402010 | Chlorpromazine 25mg/ml injection |
| 840 | 0402010 | Fentazin 2mg tablets (AMCo) |
| 8445 | 0402010 | Stelabid Tablet (GlaxoSmithKline Consumer Healthcare) |
| 8506 | 0402010 | Chlorpromazine 100mg suppository |
| 8519 | 0402010 | Chlorpromazine 100mg/5ml oral solution |
| 8537 | 0402010 | Trifluoperazine 1mg/ml Injection |
| 8637 | 0402010 | Pimozide 10mg tablet |
| 8712 | 0402010 | Flupentixol decanoate 100mg/ml Injection |
| 8771 | 0402010 | Largactil 10mg Tablet (Hawgreen Ltd) |
| 8881 | 0402010 | Remoxipride 150mg capsule |
| 8903 | 0402010 | Sulpiride 200mg/5ml oral solution sugar free |
| 8921 | 0402010 | Integrin 10mg Capsule (Sanofi-Synthelabo Ltd) |
| 8979 | 0402010 | Serenace 1.5mg tablets (Teva UK Ltd) |
| 8985 | 0402010 | Stelazine 1mg/5ml syrup (Mercury Pharma Group Ltd) |
| 9022 | 0402010 | Fluphenazine decanoate 25mg/ml Injection |
| 9190 | 0402010 | Chlorpromazine 25mg/5ml oral solution sugar free |
| 9247 | 0402010 | Sulpiride 400mg tablets |
| 9340 | 0402010 | Risperdal 3mg tablets (Janssen-Cilag Ltd) |
| 9347 | 0402010 | Clopixol 25mg tablets (Lundbeck Ltd) |
| 9475 | 0402010 | Risperdal 4mg tablets (Janssen-Cilag Ltd) |
| 9515 | 0402010 | Zoleptil 50 tablets (Movianto UK Ltd) |
| 9659 | 0402010 | Risperdal 2mg tablets (Janssen-Cilag Ltd) |
| 9686 | 0402010 | Zuclopenthixol 10mg tablets |
| 9794 | 0402010 | Quetiapine 200mg tablets |
| 9975 | 0402010 | Haloperidol 1mg/ml sugar free Oral solution |

Source: Stocks et al (8)

## Medication review codes

| **Med code** | **Read code** | **Description** |
| --- | --- | --- |
| 109737 | 8BM0200 | Dementia medication review |
| 1665 | 8B31400 | Medication review |
| 8034 | 8B3S.00 | Medication review |
| 106410 | 8B31B00 | Polypharmacy medication review |
| 12741 | 8BIC.00 | Medication review done by pharmacist |
| 69681 | 8BMH.00 | Medication review done by pharmacy technician |
| 30435 | 8BIH.00 | Medication review done by doctor |
| 11258 | 8BI..00 | Other medication review |
| 19157 | 8BIy.00 | Medication review done by nurse |
| 104551 | 8BT2.00 | Medication review done by practice nurse |
| 11955 | 8B3V.00 | Medication review done |
| 11843 | 8B3x.00 | Medication review with patient |
| 12589 | 8B3h.00 | Medication review without patient |
| 11258 | 8BI..00 | Other medication review |
| 101026 | 8BT..00 | Medication review - additional |
| 102421 | 8BMY.00 | Medication review done by medicines management pharmacist |
| 102815 | 8BMX.00 | Medication review done by medicines management technician |
| 103676 | 8BM0100 | Antipsychotic medication review |

Source: CPRD GOLD Code Browser Version 3.0.0 (2)

## Primary care appointments post-hospital discharge codes

| **Consultation code** | **Consultation type** | Routine or acute appointment |
| --- | --- | --- |
| 1 | Clinic | Routine |
| 2 | Night visit, deputising service | Acute |
| 3 | Follow-up/routine visit | Routine |
| 4 | Night visit, local rota | Acute |
| 6 | Night visit, practice | Acute |
| 7 | Out of hours, practice | Acute |
| 9 | Surgery consultation | Routine |
| 11 | Acute visit | Acute |
| 18 | Emergency consultation | Acute |
| 21 | Telephone call to a patient | Routine |
| 27 | Home visit | Routine |
| 30 | Nursing Home visit | Routine |
| 31 | Residential Home visit | Routine |
| 32 | Twilight visit | Routine |
| 35 | Co-op telephone advice | Acute |
| 36 | Co-op surgery consultation | Acute |
| 37 | Co-op home visit | Acute |
| 40 | Community clinic | Routine |
| 50 | Night visit | Acute |
| 55 | Telephone consultation | Routine |
| 61 | Extended hours | Routine |

Source: CPRD GOLD database (2)

## Residential status codes

| **Med code** | **Read code** | **Description** |
| --- | --- | --- |
| 13359 | 13F6100 | Lives in a nursing home |
| 13360 | 13F6.00 | Nursing/other home |
| 24828 | Z177F00 | Nursing home care |
| 73321 | 9b1P.00 | Nursing home |
| 24816 | Z177C00 | Residential care |
| 24956 | 13FK.00 | Lives in a residential home |
| 53140 | Z177D00 | Local authority residential care |
| 59548 | 13FT.00 | Lives in an old people’s home |
| 11419 | 13F7200 | Lives in an old people’s home |
| 46642 | 9Bb79.00 | Other residential care homes managed by local authority |
| 49681 | 13FX.00 | Lives in care home |

Source: CPRD GOLD Code Browser Version 3.0.0 (2)

## Body mass index and weight codes

| **Med code** | **Read code** | **Description** | **Category** |
| --- | --- | --- | --- |
| 912 | R030.00 | [D]Anorexia | Underweight |
| 32914 | 22K3.00 | Body Mass Index low K/M2 | Underweight |
| 2135 | E271.00 | Anorexia nervosa | Underweight |
| 126 | 22A6.00 | O/E - Underweight | Underweight |
| 24496 | 22K6.00 | Body mass index less than 20 | Underweight |
| 12530 | R034800 | [D]Underweight | Underweight |
| 30570 | Eu50000 | [X]Anorexia nervosa | Underweight |
| 53746 | R030z00 | [D]Anorexia NOS | Underweight |
| 34929 | Eu50100 | [X]Atypical anorexia nervosa | Underweight |
| 28946 | 22K1.00 | Body Mass Index normal K/M2 | Healthy weight |
| 44291 | 22K8.00 | Body mass index 20-24 - normal | Healthy weight |
| 23376 | 22A3.00 | O/E - weight within 10% ideal | Healthy weight |
| 43472 | 66CK.00 | Target weight reached | Healthy weight |
| 52703 | 212Q.00 | Obesity resolved | Healthy weight |
| 107231 | 22KA.00 | Target body mass index | Healthy weight |
| 9015 | 22K4.00 | Body mass index index 25-29 - overweight | Overweight |
| 2839 | 22A4.11 | O/E - overweight | Overweight |
| 16404 | 22A4.00 | O/E - weight 10-20% over ideal | Overweight |
| 32974 | 22A5.00 | O/E - weight > 20% over ideal | Overweight |
| 103499 | 22AA.00 | Overweight | Overweight |
| 102150 | 66CM.00 | Risk health associ overweight and obesity, at increased risk | Overweight |
| 102514 | 66CN.00 | Risk health associated overweight and obesity, at high risk | Overweight |
| 104724 | 66CL.00 | Risk health associa overweight obesity, at no increased risk | Overweight |
| 430 | C380.00 | Obesity | Obese |
| 28937 | 22K2.00 | Body Mass Index high K/M2 | Obese |
| 13278 | 22K5.00 | Body mass index 30+ - obesity | Obese |
| 11461 | 66C..00 | Obesity monitoring | Obese |
| 22556 | 22K7.00 | Body mass index 40+ - severely obese | Obese |
| 8854 | C380300 | Morbid obesity | Obese |
| 22695 | C380400 | Central obesity | Obese |
| 11401 | C38z000 | Simple obesity NOS | Obese |
| 66406 | C38..00 | Obesity and other hyperalimentation | Obese |
| 38059 | C380200 | Extreme obesity with alveolar hypoventilation | Obese |
| 38799 | C380000 | Obesity due to excess calories | Obese |
| 25968 | C380500 | Generalised obesity | Obese |
| 24755 | C38y.11 | Pickwickian syndrome | Obese |
| 103574 | C38y011 | Obesity hypoventilation syndrome | Obese |
| 38294 | C38y000 | Pickwickian syndrome | Obese |
| 49250 | C380100 | Drug-induced obesity | Obese |
| 69757 | Cyu7000 | [X]Other obesity | Obese |
| 70898 | C38z.00 | Obesity and other hyperalimentation NOS | Obese |
| 104421 | C380700 | Lifelong obesity | Obese |
| 52782 | Cyu7.00 | [X]Obesity and other hyperalimentation | Obese |
| 104129 | C380600 | Adult-onset obesity | Obese |

Source: CPRD GOLD Code Browser Version 3.0.0 (2)

References to sources:

1. Kontopantelis E, Springate D, Reeves D, Ashcroft DM, Valderas JM, Doran T. Withdrawing performance indicators: retrospective analysis of general practice performance under UK Quality and Outcomes Framework. BMJ. 2014;348:g330.

2. Clinical Practice Research Datalink. CPRD GOLD: Medicines & Healthcare products Regulatory Agency; 2024 [Available from: <https://www.cprd.com/data/primary-care-data/cprd-gold>.

3. Kuan V, Denaxas S, Gonzalez-Izquierdo A, Direk K, Bhatti O, Husain S, et al. A chronological map of 308 physical and mental health conditions from 4 million individuals in the English National Health Service. The Lancet Digital Health. 2019;1(2):e63-e77.

4. Wright AK, Kontopantelis E, Emsley R, Buchan I, Sattar N, Rutter MK, et al. Life expectancy and cause-specific mortality in type 2 diabetes: a population-based cohort study quantifying relationships in ethnic subgroups. Diabetes care. 2017;40(3):338-45.

5. Metcalfe D, Masters J, Delmestri A, Judge A, Perry D, Zogg C, et al. Coding algorithms for defining Charlson and Elixhauser co-morbidities in Read-coded databases. BMC medical research methodology. 2019;19:1-9.

6. Kontopantelis E, Olier I, Planner C, Reeves D, Ashcroft DM, Gask L, et al. Primary care consultation rates among people with and without severe mental illness: a UK cohort study using the Clinical Practice Research Datalink. BMJ Open. 2015;5(12):e008650.

7. Hoile R, Tabet N, Smith H, Bremner S, Cassell J, Ford E. Are symptoms of insomnia in primary care associated with subsequent onset of dementia? A matched retrospective case-control study. Aging & Mental Health. 2020;24(9):1466-71.

8. Stocks SJ, Kontopantelis E, Webb RT, Avery AJ, Burns A, Ashcroft DM. Antipsychotic prescribing to patients diagnosed with dementia without a diagnosis of psychosis in the context of national guidance and drug safety warnings: longitudinal study in UK general practice. Drug Safety. 2017;40:679-92.

# Table S3: Variable definitions and sources

|  | **Definition** | **Source** |
| --- | --- | --- |
| ***Demographics*** |  |  |
| **Age at index hospital admission**  **(continuous)** | Calculated on the difference between year of index hospitalisation and year of birth | Primary care data - CPRD GOLD   - Patient file |
| **Age at index hospital admission**  **(categorical)** | Calculated on the difference between year of index hospitalisation and year of birth  Categorised into 5-yearly intervals  (65-69, 70-74, 75-79, 80-84,  85-90, 90+)  Reference category: 65-69 | Primary care data - CPRD GOLD   - Patient file |
| **Gender** | Binary variable  (Male/Female)  Reference category: Male | Primary care data - CPRD GOLD   - Patient file |
| **Ethnicity** | Categorical variable  (White, Black, Asian, Mixed, Other)  ‘Unknown’ category defined as missing data  Reference category: White | Primary care data - CPRD GOLD   - Clinical file   Linked data   - HES APC |
| **Region** | Categorial variable  Geographical location of GP practice in England:  North East, North West, Yorkshire & The Humber, East Midlands, West Midlands, East of England, South West, South Central, London, South East Coast  Reference category: South West (region with the most patients) | Primary care data - CPRD GOLD   - Practice file |
| **GP Index of Multiple Deprivation (IMD)** | Categorical variable  Quintile IMD scores of GP practice  1 = Least deprived  5 = Most deprived  IMD based on geography from UK Census in England  Reference category: 5 (Most deprived) | Linked data  Most recent IMD score   - CPRD GOLD practice IMD file |
| **Residential care** | Binary variable  (no/yes)  Coded for living in a nursing home, residential home or care home | Primary care data - CPRD GOLD  Read code any time from dementia diagnosis   - Clinical file |
| ***Clinical*** |  |  |
| **Dementia diagnosis** | Whole cohort  Earliest record dementia diagnosis or dementia drug prescription | Primary care data - CPRD GOLD  First recorded Read code in   - Clinical file   First recorded product or BNF code in   - Therapy file |
| **Dementia type** | Categorical variable  (Alzheimer’s, Vascular dementia, Dementia with Lewy Bodies, Mixed, Alcoholic, Unspecified)  Reference category: Unspecified | Primary care data - CPRD GOLD  linked with first recorded dementia diagnosis   - Clinical file |
| **Body Mass Index (BMI)**  **(kg/m^2^)** | Categorical variable  Underweight (<18.5)  Healthy weight (18.5-24.9)  Overweight (25-29.9)  Obese (≥30)  Reference category: Healthy weight | Primary care data - CPRD GOLD  Most recent BMI record from   - Clinical file - Additional file (height and weight measurements) |
| **COPD** | Binary variable  (no/yes)  Ever coded for COPD | Primary care data - CPRD GOLD  At least one Read code any time from dementia diagnosis in either   - Clinical file - Referral file |
| **Cardiovascular Disease** | Binary variable  (no/yes)  Ever coded for cardiovascular disease | Primary care data - CPRD GOLD  At least one Read code any time from dementia diagnosis in either   - Clinical file - Referral file |
| **Heart Failure** | Binary variable  (no/yes)  Ever coded for heart failure | Primary care data - CPRD GOLD  At least one Read code any time from dementia diagnosis in either   - Clinical file - Referral file |
| **Chronic Kidney Disease** | Binary variable  (no/yes)  Ever coded for chronic kidney disease | Primary care data - CPRD GOLD  At least one Read code any time from dementia diagnosis in either   - Clinical file - Referral file |
| **Diabetes Mellitus (type 1 or 2)** | Binary variable  (no/yes)  Ever coded for type 1 or type 2 diabetes | Primary care data - CPRD GOLD  At least one Read code any time from dementia diagnosis in either   - Clinical file - Referral file |
| **Cerebrovascular Disease** | Binary variable  (no/yes)  Ever coded for cerebrovascular disease (excluding stroke-related conditions) | Primary care data - CPRD GOLD  At least one Read code any time from dementia diagnosis in either   - Clinical file - Referral file |
| **History of Stroke** | Binary variable  (no/yes)  Ever coded for a history of stroke | Primary care data - CPRD GOLD  At least one Read code any time from dementia diagnosis in either   - Clinical file - Referral file |
| **Depression** | Binary variable  (no/yes)  Ever coded for depression | Primary care data - CPRD GOLD  At least one Read code any time from dementia diagnosis in either   - Clinical file - Referral file |
| **Anxiety** | Binary variable  (no/yes)  Ever coded for anxiety | Primary care data - CPRD GOLD  At least one Read code any time from dementia diagnosis in either   - Clinical file - Referral file |
| **Insomnia** | Binary variable  (no/yes)  Ever coded for insomnia | Primary care data - CPRD GOLD  At least one Read code any time from dementia diagnosis in either   - Clinical file - Referral file |
| **Long-term conditions (LTC) category** | Categorical variable  (0, 1, 2-3, 4-5, 6+ LTCs)  Reference category: 0  LTCs = COPD, cardiovascular disease, heart failure, chronic kidney disease, type 1 or type 2 diabetes mellitus, cerebrovascular disease, history of stroke, depression, anxiety, insomnia. | Primary care data - CPRD GOLD  At least one Read code any time from dementia diagnosis in either   - Clinical file - Referral file |
| **Polypharmacy** | Binary variable  (no/yes)  At least 5 different prescribed medications within a 90-day period, between dementia diagnosis date and index hospitalisation date | Primary care data - CPRD GOLD  At least 5 BNF codes   - Therapy file |
| **Antipsychotic medication** | Binary variable  (no/yes)  At least 2 prescriptions for antipsychotic medication within a 12-month period, between dementia diagnosis date and index hospitalisation date | Primary care data - CPRD GOLD  At least 2 product codes   - Therapy file |
| **Medication reviews** | Binary variable  (no/yes)  At least one medication review 12-months before index hospitalisation date | Primary care data - CPRD GOLD   - Clinical file |
| **Primary care appointment within 2 weeks of index discharge** | Binary variable  (no/yes)  Categorical variable  (routine visit/acute visit/no visit)  Reference category: no visit  Follow-up primary care appointment within two weeks of index hospital discharge | Primary care data - CPRD GOLD   - Consultation file |
| **Index hospital admission** | Whole cohort  Record of first hospital admission any time after dementia diagnosis | Linked data   - HES APC |
| **180-day hospital readmission** | Binary variable  (no/yes)  Record of second hospital admission that is maximum 180 days after index hospital admission | Linked data   - HES APC |
| **Follow-up (days)** | Continuous variable  Days from index hospital admission to first occurrence of last data collection date from GP, GP transfer-out date, death date, or survival until end of study period | Primary care data - CPRD GOLD   - Patient file - Practice file   Linked data   - HES APC |

CPRD GOLD = Clinical Practice Research Datalink GOLD, HES APC = Hospital Episode Statistics Admitted Patient Care, COPD = Chronic Obstructive Pulmonary Disease

# S4: Data preparation and analyses code

All code is available via the GitHub link: [Bria-b/CPRD-dementia-readmissions: Python and R scripts for defining variables and data analysis from a CPRD database](https://github.com/Bria-b/CPRD-dementia-readmissions)

# Table S5: Sensitivity analysis examining primary care visit type and hospital readmission

| Variable  (Reference category) | Odds ratio  (95% confidence interval) | p-value |
| --- | --- | --- |
| **Primary care appointment after discharge** (no visit) |  |  |
| Acute visit | 1.53 (1.36-1.73) | <0.001* |
| Routine visit | 1.20 (1.14-1.25) | <0.001* |

*Significant p-value set at p<0.05

# Table S6: Interaction analysis between antipsychotic prescribing and hospital admission year group

| **Model comparison** | **ꭓ^2^** | **df** | **p-value** |
| --- | --- | --- | --- |
| Interaction vs no interaction | 4.59 | 4 | 0.34 |

# Table S7: Stratified logistic regression analyses examining the association between antipsychotic prescribing and hospital readmission by residential status

| **Residential status** | **OR (95% confidence interval)** | **p-value** |
| --- | --- | --- |
| Residential care | 0.95 (0.72-1.26) | 0.75 |
| Non-residential care | 0.91 (0.84-0.98) | 0.01* |

*Significant p-value set at p<0.05

# Table S8: Association between antipsychotic medication prescribing and hospital readmission among patients diagnosed with dementia across the full study period (1997–2018) and after 2009

| **Antipsychotic medication** | **OR (95% confidence interval)** | **p-value** |
| --- | --- | --- |
| For patients diagnosed with dementia across study period (1997-2018) | 0.91 (0.85-0.98) | 0.01* |
| For patients diagnosed with dementia after 2009 | 1.06 (0.91-1.23) | 0.48 |

*Significant p-value set at p<0.05
